# The LytS-type histidine kinase BtsS is a 7-transmembrane receptor that binds pyruvate

**DOI:** 10.1128/mbio.01089-23

**Published:** 2023-09-01

**Authors:** Jin Qiu, Ana Gasperotti, Nathalie Sisattana, Martin Zacharias, Kirsten Jung

**Affiliations:** 1 Faculty of Biology, Microbiology, Ludwig-Maximilians-Universität München, Martinsried, Germany; 2 Center of Functional Protein Assemblies, Technical University of Munich, Garching, Germany; Instituto Gulbenkian de Ciência, Oeiras, Portugal

**Keywords:** LytS/LytTR, receptor, histidine kinase, response regulator, bacterial sensing and signal transduction, stimulus perception

## Abstract

LytS/LytTR-type histidine kinase/response regulator systems regulate crucial host-specific mechanisms during infection of human or plant hosts. *Escherichia coli* has two of them, and the histidine kinase BtsS is a high-affinity sensor for extracellular pyruvate that together with the response regulator BtsR activates the expression of *btsT,* which encodes a high-affinity pyruvate transporter. However, the molecular mechanism of how pyruvate binding triggers a response is still unclear. Here, we experimentally show that BtsS consists of seven transmembrane helices, with the N-terminus exposed on the periplasmic side. Using a site-directed mutagenesis-based screening assay, Arg72, Arg99, Cys110, and Ser113, all located on the periplasmic side in three transmembrane helices, were identified as critical for the binding of pyruvate to BtsS, a finding that was further confirmed by molecular dynamics simulation studies. In addition, Mn^2+^-dependent autophosphorylation of wild-type BtsS was demonstrated for the first time, and individual replacement of the four amino acids affected this process as well as dimerization and consequently *btsT* expression. This study demonstrates how binding of a metabolite to the membrane-integrated sensor domain triggers signaling in the cytoplasm.

**IMPORTANCE:**

Here, we studied the LytS-type histidine kinase BtsS of *E. coli* and identified the pyruvate binding site within the membrane-spanning domains. It is a small cavity, and pyruvate forms interactions with the side chains of Arg72, Arg99, Cys110, and Ser113 located in transmembrane helices III, IV, and V, respectively. Our results can serve as a starting point to convert BtsS into a sensor for structurally similar ligands such as lactate, which can be used as biosensor in medicine.

## INTRODUCTION

Two-component systems (TCSs) in most cases consist of a membrane-anchored histidine kinase (HK) and a cognate cytoplasmic response regulator (RR) (1). All histidine kinases have two conserved subdomains: the dimerization and histidine phosphotransfer (DHp) domain (2), which includes a conserved histidine for phosphorylation, and the catalytic and ATP-binding (CA) domain, which binds ATP and catalyzes the transfer of the γ-phosphoryl group to the histidine (3). Among the 30 TCSs of *Escherichia coli,* there are two LytS/LytTR-type systems, namely, BtsS/BtsR (4, 5) and YpdA/YpdB (6) (recently renamed as PyrS/PyrR [7]), both of which are involved in pyruvate sensing. The BtsS/BtsR system is widely distributed in γ-proteobacteria, and many LytS/LytTR-type systems regulate crucial host-specific mechanisms during infection of human or plant hosts (8, 9).

**Fig 1:**
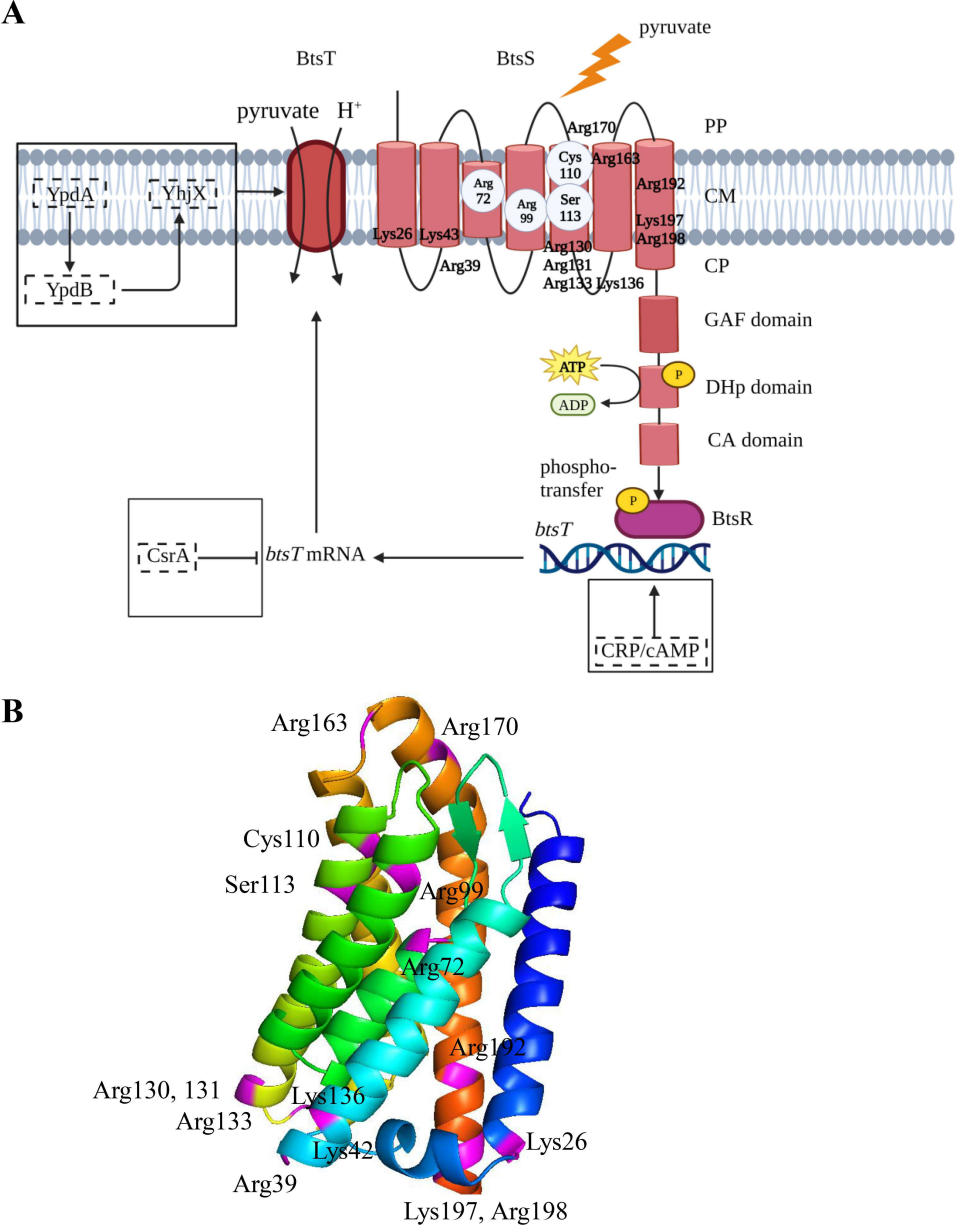
The pyruvate-responsive histidine kinase/response regulator BtsS/BtsR system of *E. coli*. (**A**) Schematic of the BtsS/BtsR signaling cascade regulating the expression of *btsT*, which encodes a high-affinity pyruvate transporter. The system is embedded in a complex regulatory network (proteins with regulatory function are shown in dashed boxes); see text for details. Based on our finding that the N-terminus of BtsS is periplasmically localized, the receptor is drawn with seven transmembrane helices. The position of all lysines and arginines in the transmembrane domain is marked. Amino acids involved in pyruvate binding, Arg72, Arg99, Cys110, and Ser113, are labeled in a gray circle. PP: periplasm, CM: cytoplasmic membrane, CP: cytoplasm. (**B**) AlphaFold2 model of the transmembrane domain of BtsS in rainbow colors, with the N-terminus colored in blue. All positively charged amino acids are labeled in purple.

BtsS consists of an input domain containing the membrane-integrated 5TMR_LYT domain (pfam07694) and the cytosolic GAF domain, and the DHp and CA domains (5) (Fig. 1; Fig. S1A). BtsR belongs to the family of LytTR-type response regulators and consists of a CheY-like receiver domain linked to a LytR-type DNA-binding domain. This DNA-binding domain is predicted to form a 10-stranded β-fold (10, 11). In previous work, we found that BtsS is a high affinity sensor for extracellular pyruvate (*K_d_* = 58.6 µM) (4). Upon sensing pyruvate, BtsS, together with BtsR, activates the expression of *btsT,* which encodes the high-affinity pyruvate/H^+^ symporter BtsT (12) (Fig. 1). Expression of *btsT* underlies catabolite repression, and additionally requires the cyclic AMP (cAMP) receptor protein (CRP) (CRP-cAMP) for expression (5). At the posttranscriptional level, *btsT* is regulated by carbon storage regulator A (CsrA) (13). In addition, *btsT* transcription is under positive feedback regulation by the YpdA/YpdB-system and its target gene product YhjX (14). The molecular mechanism of how pyruvate binding triggers a response in the BtsS/BtsR system is still unclear.

It should be noted that *E. coli* has two different LytS/LytTR-type systems for pyruvate sensing (BtsS/BtsR and PyrS/PyrR) and three pyruvate transporters (BtsT, YhjX, and CstA) (15), whereas the pathogen *Salmonella enterica* serovar Typhimurium has one pyruvate sensing system (BtsS/BtsR) and two pyruvate transporters (BtsT and CstA) (16), and the marine pathogen *Vibrio campbellii* has one pyruvate sensing system (BtsS/BtsR) and only one pyruvate transporter (BtsU) (17). These bacteria not only possess different numbers and types of pyruvate sensing and uptake systems, but they also differ phenotypically when pyruvate uptake is prevented by deletion of the corresponding transporter genes, especially when infecting their respective hosts (16 – 18).

Here, we showed that the LytS-type histidine kinase BtsS of *E. coli* is a sensor with the N-terminus located on the periplasmic side. We found that Arg72, Arg99, Cys110, and Ser113 are crucial for the binding of pyruvate to BtsS and subsequent autophosphorylation, and consequently *btsT* expression. For the first time, we demonstrated the autophosphorylation activity of BtsS, and this LytS-type histidine kinase was found to prefer Mn^2+^-ATP instead of Mg^2+^-ATP.

## RESULTS

### Periplasmic location of the N-terminus of BtsS

**Fig 2:**
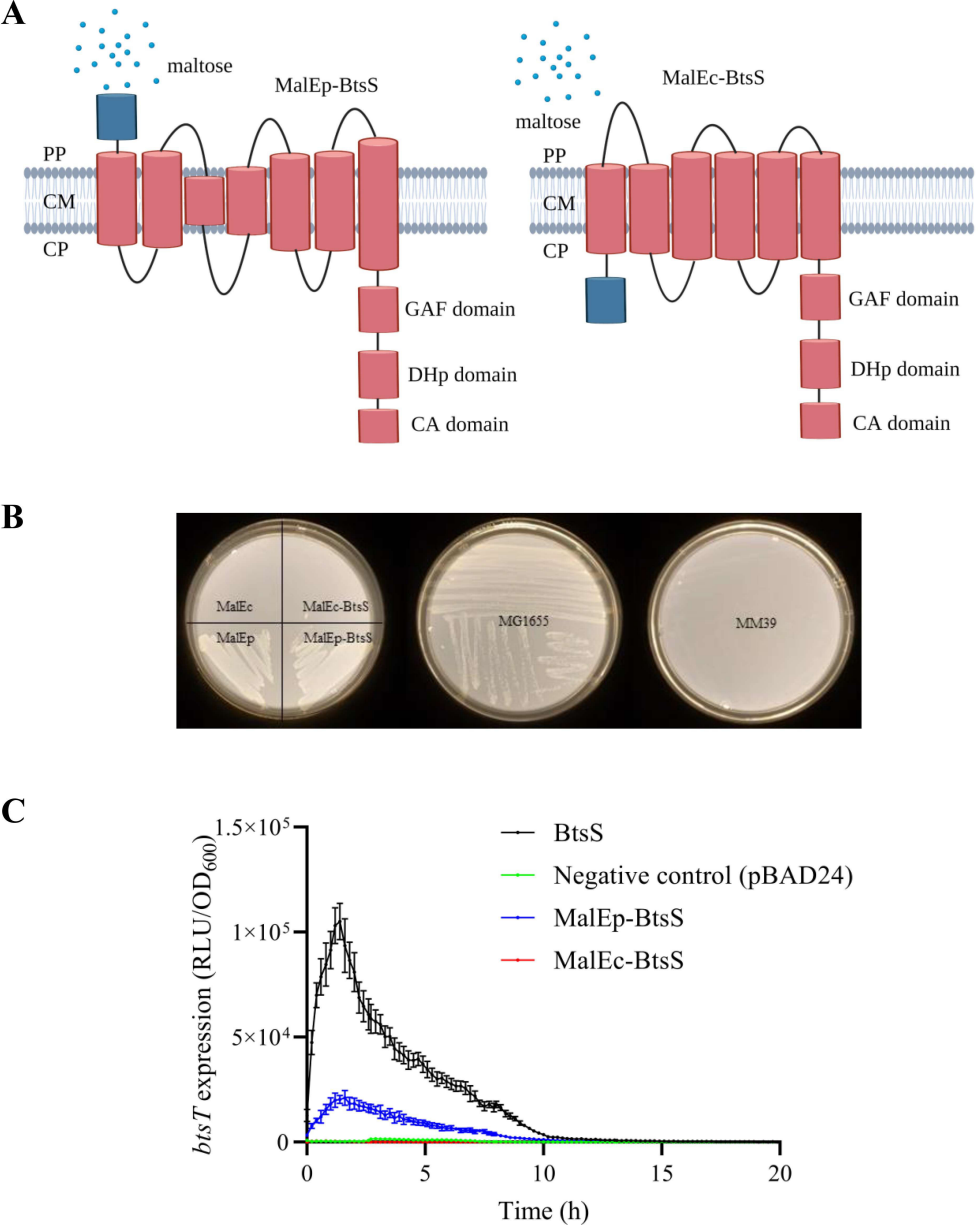
Periplasmic location of the N-terminus of BtsS. (**A**) Schematic illustration of the use of maltose-binding protein MalE (blue-labeled domain) as reporter protein to test the location of the N-terminus of BtsS. MalE is a periplasmic protein that binds maltose, which is imported by the ABC transporter complex MalEFGK. See text for more details. (**B**) Growth of *E. coli* MM39 (∆*malE*) after complementation with different MalE and MalE-BtsS variants on maltose as sole C source compared to *E. coli* MG1655 (*malE* ^+^) and MM39 (∆*malE*). (**C**) Functionality of MalE-BtsS variants was tested using the reporter strain MG1655∆*btsSR/*pBBR-*btsT-lux* transformed either with pBAD24 as a negative control, pBAD24-*btsS/R* as a positive control, pBAD24-pMALp-*btsS/R* or pBAD24-pMALc-*btsS/R*. Cells of an overnight culture were inoculated in M9 minimal medium containing pyruvate (5 mM) and succinate (15 mM) as C source and grown at 37°C in a microplate reader. OD_600_ and luminescence were measured, and data are relative light units (RLU) in counts per second per OD. The time course of BtsS/BtsR-mediated *btsT* expression (measured as P*_btsT_::luxCDABE* and reported in relative light units) was measured and shown. All experiments were performed as biological triplicates (*n* = 3), and representative plates are shown. Error bars represent the standard deviation of the means.

BtsS is a LytS-type histidine kinase. The characteristic feature of all members of the LytS-type family is the 5TMR-LYT domain suggesting that BtsS is anchored with at least five transmembrane helices in the membrane. However, the first 36 amino acids of the receptor, which form at least another transmembrane helix, do not belong to this domain (Fig. S1A). Prediction of transmembrane helices based on hydrophobicity using three software tools (TMHMM-2.0 [19, 20], ProtScale [21], and PsiPred [22]) (Fig. S1B through D) led to the model that BtsS has six transmembrane helices and an N-terminus oriented toward the cytoplasm (Fig. 2A, right panel). The 3D model predicted by AlphaFold2 (23) described BtsS (uniprot ID: P0AD14) with seven transmembrane helices and positioned the N-terminus toward the periplasm (Fig. 1 and 2A, left panel). AlphaFold2 generates a per-residue confidence score (pLDDT9) on a scale of 0 to 100. For transmembrane helices 2–7 (starting at Val47), this score was very high (<90); for transmembrane helix 1, it was slightly lower (pLDDT9 score between 90 and 70). Therefore, it was necessary to determine the localization of the N-terminus experimentally.

To accomplish this task, we used an MalE fusion strategy that we had previously used to successfully localize the N-terminus of the quorum-sensing hybrid histidine kinase LuxN of *Vibrio harveyi* to the periplasmic site (24). MalE is a periplasmic-binding protein. It binds maltose and higher maltodextrins such as maltotriose, which are imported by the ABC transporter complex MalEFGK. Wild-type MalE (designated as MalEp and encoded by the commercially available vector pMAL-p2X) contains a leader sequence to translocate the protein through the cytoplasmic membrane via the Sec system. In contrast, MalEc (encoded by the commercially available vector pMAL-c2X), which lacks this leader sequence is produced as a cytoplasmic protein (25). Both *malE* variants were fused to the 5′ end of *btsS* and encode hybrid proteins that are supposed to have either periplasmically localized (MalEp-BtsS) or cytoplasmically localized (MalEc-BtsS) maltose-binding protein (MBP) (Fig. 2A). We then tested the position of MalE in the hybrid proteins by complementation of a ∆*malE* mutant (*E. coli* MM39). Unlike *E. coli* MG1655 the *E. coli* mutant MM39 has a deletion of the *malE* gene and cannot grow with maltose as the sole carbon source (C source) (Fig. 2B). Subsequently, *E. coli* MM39 was transformed with plasmids pMAL-p2X or pMALp-*btsS* expressing periplasmic MalE (MalEp) or the hybrid MalEp-BtsS, respectively. Both strains were able to grow with maltose as a C source, indicating functional active MalE. In contrast, *E. coli* strain MM39 transformed with pMAL-c2X or pMALc-*btsS* encoding MalE without leader peptide (MalEc) or the hybrid MalEc-BtsS, respectively, was unable to grow on maltose-containing plates (Fig. 2B).

We next tested the functionality of each MBP-BtsS construct by monitoring BtsS/BtsR signal transduction *in vivo*. We assumed that only the hybrid protein with the N-terminus of BtsS on the correct side of the membrane would result in a correct membrane topology and a functional active receptor. The MBP-BtsS constructs were tested together with the response regulator BtsR for complementation of our reporter strain lacking the *btsS/R* genes and expressing a *btsT* promoter-luciferase fusion (P*_btsT_::luxCDABE*). Strains were cultivated in minimal medium with pyruvate (5 mM) and succinate (15 mM) as C source, a condition that induces *btsT* expression (4, 9). We found that MalEp-BtsS mediated pyruvate-dependent activation of *btsT* expression similarly to wild-type BtsS, but the level of induction was reduced 5.1-fold (Fig. 2C). In contrast, MalEc-BtsS was unable to induce *btsT* expression (Fig. 2C). These results indicate that only fusion of the sequence encoding MalE with a leader peptide to the 5′-end of *btsS* resulted in a hybrid protein with a functional MalE in the periplasm and a signaling active BtsS. In contrast, fusion of *malE* without the sequence encoding a leader peptide to *btsS* forced MalE into the cytoplasm and resulted in an inactive BtsS (Fig. 2C). These results support a periplasmic location of the N-terminus of BtsS, implying that this receptor is anchored in the cytoplasmic membrane with seven transmembrane domains (Fig. 1 and 2A, left panel).

### Screening for amino acids involved in pyruvate sensing by alanine scanning mutagenesis

Our previous work has shown that BtsS is a high-affinity pyruvate receptor (*K_d_* = 58.6 µM) and that pyruvate binds to the extracellular side of the membrane-spanning domain (4). Here, our goal was to identify the pyruvate-binding site. Pyruvate has a carboxyl group that is in contact with positively charged amino acids in other pyruvate-binding proteins, such as KinD (26), or the pyruvate formate lyase (27).

**Fig 3:**
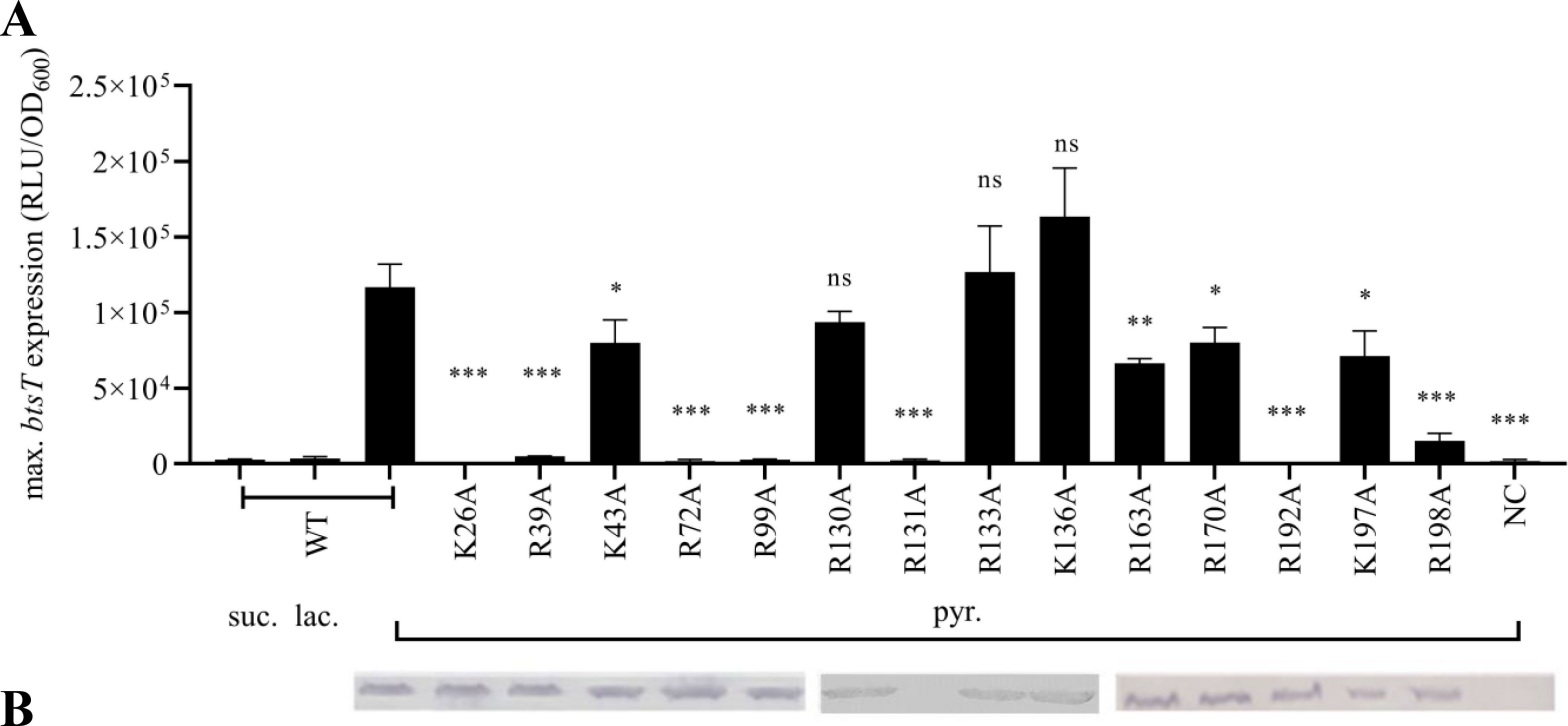
Screening for amino acids involved in pyruvate sensing in BtsS. (**A**) Reporter strain MG1655∆*btsSR* encoding the promoter fusion P*_btsT_::luxCDABE* was transformed either with plasmid pBAD24 as a negative control (NC), pBAD24-*btsS/R* (WT) as a positive control, or pBAD24-*btsS/R* encoding BtsS variants. The time course of BtsS/BtsR-mediated *btsT* expression (measured as P*_btsT_::luxCDABE* and reported in relative light units) was measured. Cells were grown with pyruvate as C source as described in Fig. 2. The activity of all BtsS variants was tested in the same way, and values of the maximal *btsT* promoter activities were reported. As negative controls, the response of WT to succinate (suc.) and lactate (lac.) was tested. The maximal luciferase activity (RLU) normalized to an optical density (OD_600_) of 1 served as the measure of *btsT* expression. All experiments were performed as biological triplicates (*n* = 3), and the error bars represent the standard deviation of the means. (**B**) Verification of production and integration of BtsS variants in the cytoplasmic membrane of *E. coli*. Cells were disrupted and fractionated, 25 µg protein of the membrane fraction was analyzed by SDS-PAGE and western blotting. BtsS was detected by a monoclonal mouse antibody against the Flag tag and an alkaline phosphatase-coupled secondary antibody. Statistics: Student’s unpaired two-sided *t*-test (***P < 0.001; **P < 0.01; *P < 0.05; ns, *P* > 0.05).

The input domain of BtsS contains ten arginines and four lysines (Fig. 1). To screen for their importance in pyruvate binding, we replaced each of these amino acids individually with alanine. Subsequently, these BtsS variants were tested together with wild-type BtsR for complementation of our reporter strain as described above (Fig. 1C). Strains were cultivated in minimal medium with pyruvate (5 mM) and succinate (15 mM) as C source, and luminescence levels of the P*_btsT_::luxCDABE* reporter were measured. Of the 14 tested variants, the variants BtsS-K26A, BtsS-R39A, BtsS-R72A, BtsS-R99A, BtsS-R131A, and BtsS-R192A were unable to induce *btsT* (Fig. 3A). Although the activity of variant BtsS-R198A was greatly reduced, this receptor still responded to increasing concentrations of pyruvate (Fig. S2A). The production and integration of all BtsS variants into the cytoplasmic membrane of *E. coli* were tested by resolving the proteins of the membrane fractions by SDS-PAGE and western blot detection of the FLAG tag. All BtsS variants except BtsS-R131A were produced and integrated into the membrane (Fig. 3B). This initial screening showed that Lys26, Arg39, Arg72, Arg99, and Arg192 might be involved in pyruvate-sensing and/or signaling of BtsS.

### Arg72, Arg99, Cys110, and Ser113 are involved in pyruvate sensing

**Fig 4:**
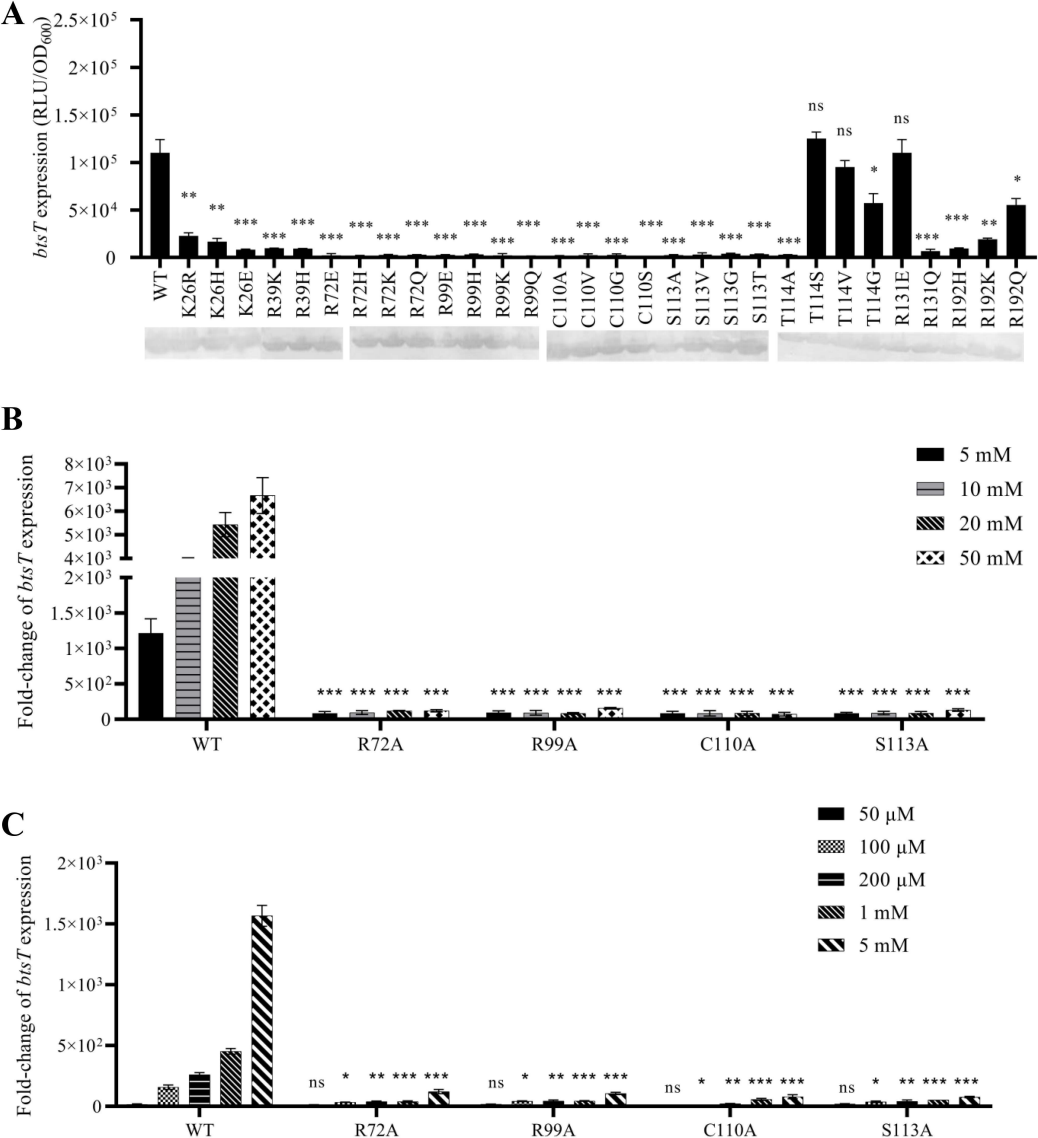
Arg72, Arg99, Cys110, and Ser113 are essential for pyruvate sensing in BtsS. (**A**) Reporter strain MG1655∆*btsSR* encoding the promoter fusion P*_btsT_::luxCDABE* was transformed either with plasmid pBAD24-*btsS/R* (WT) as a positive control, or pBAD24-*btsS/R* encoding BtsS variants. The time course of BtsS/BtsR-mediated *btsT* expression (measured as P*_btsT_::luxCDABE* and reported in relative light units) was measured. Cells were grown with 5 mM pyruvate and 15 mM succinate as C source as described in Fig. 2. The activity of all BtsS variants was tested in the same way, and values of the maximal *btsT* promoter activities were reported. Maximum luciferase activity (RLU), normalized to an optical density (OD_600_) of 1, served as a measure of *btsT* expression. (**B**) Using the same protocol, different concentrations of pyruvate (5 mM, 10 mM, 20 mM, and 50 mM) were tested as the C source, with the total C concentration kept constant at 50 mM in each case by addition of succinate. Fold-change values were calculated using the succinate control. (**C**) Alternatively, overnight grown cells as in A and B were inoculated in 0.1× LB medium and after 1 h, the indicated concentration of pyruvate or water (negative control) was added and growth and luminescence were followed. Cells immediately induced *btsT* expression. Again, values of the maximal *btsT* promoter activities were reported. Fold-change values were calculated using the water control. All experiments were performed as biological triplicates (*n* = 3), and the error bars represent the standard deviation of the means. Statistics: Student’s unpaired two-sided *t*-test (***P < 0.001; **P < 0.01; *P < 0.05; ns, *P* > 0.05).

Substitution of a positively charged amino acid by alanine not only removes the charged side chain but also significantly reduces its size. Therefore, all amino acids identified as crucial by the alanine scanning approach were further replaced by amino acids selected to maintain the charge (Arg, Lys, His) and size (Gln) or even to have the opposite charge (Glu). Arg72 and Arg99 could not be replaced by any other amino acid because all corresponding BtsS variants prevented *btsT* induction (Fig. 4A). These amino acids appear to be essential for stimulus perception or signal transduction of BtsS.

In contrast, replacement of Lys26, Arg39, Arg131, and Arg192 with positively charged amino acids and other amino acids was tolerated, and the corresponding BtsS variants were able to respond to pyruvate (Fig. 4A). Consistent with the “positive-inside” rule (28), positively charged amino acids at the cytoplasmic end of the transmembrane helices, e.g., at positions 26, 39, and 131 (Fig. 1), could be important for the efficiency of insertion of the receptor into the membrane. In contrast, Arg192 is located in the hydrophobic core of the membrane (Fig. 1) and could be replaced by Lys or Gln but not by Ala (Fig. 3 and 4A). As described below, BtsS-R192A had comparable pyruvate-stimulable autokinase activity to wild type *in vitro* (Fig. S9). Therefore, we did not investigate these four amino acids further.

We then searched for amino acids that might be in contact with the hydroxyl group of pyruvate. We focused on amino acids near Arg72 and Arg99 in the structural model of BtsS. Therefore, we replaced Asn70, Thr71, Thr94, Tyr100, Ser101, Thr106, Cys110, Ser113, and Thr114 with Ala. The BtsS variants N70A, T71A, T94A, Y100A, S101A, T106A (Fig. S2B) allowed pyruvate-dependent *btsT* induction, whereas the BtsS variants C110A, S113A, and T114A were unable to do so (Fig. 4A). We then replaced Cys110, Ser113, and Thr114 with additional amino acids. All Cys110 and Ser113 BtsS variants (C110A, C110V, C110G, C110S, S113A, S113V, S113G, S113T) prevented *btsT* expression under pyruvate-inducing conditions. In contrast, BtsS variants with an exchange at the Thr114 position with Ser, Val, and Gly were active (Fig. 4A). All variants were produced as membrane-integrated proteins as verified by SDS-PAGE and western blotting. These results indicate that Cys110 and Ser113 are also essential for pyruvate sensing by BtsS.

Determination of the *in vivo* activity of the BtsS variants was thus far based on the response of the complemented reporter strain to pyruvate (5 mM) as a C source. To test for a possible change in the affinity of the BtsS variants for pyruvate, we tested the pyruvate-dependent *btsT* induction in two additional *in vivo* assays. First, strains were grown in M9 minimal medium with different pyruvate concentrations (5–50 mM), while the total carbon concentration (50 mM) was kept constant by adding succinate. Whereas we found concentration-dependent *btsT* expression for the wild-type BtsS, BtsS-R72A, BtsS-R99A, BtsS-C110A, and BtsS-S113A did not induce *btsT* expression under any condition (Fig. 4B). Our second assay was based on our previous work (4) in which we showed that starving *E. coli* cells exposed to very low pyruvate concentrations (10 µM to 1 mM) induce *btsT* in a concentration-dependent manner. We used this protocol and found not only an approximately 100-fold lower response of the BtsS-R72A, BtsS-R99A, BtsS-C110A, and BtsS-S113A variants compared with wild type, but also a slight shift to higher pyruvate concentrations (Fig. 4C). Taken together, these results suggest that Arg72, Arg99, Cys110, and Ser113 are involved in pyruvate sensing.

### Arg72, Arg99, Cys110, and Ser113 form a pyruvate-binding site

**Fig 5:**
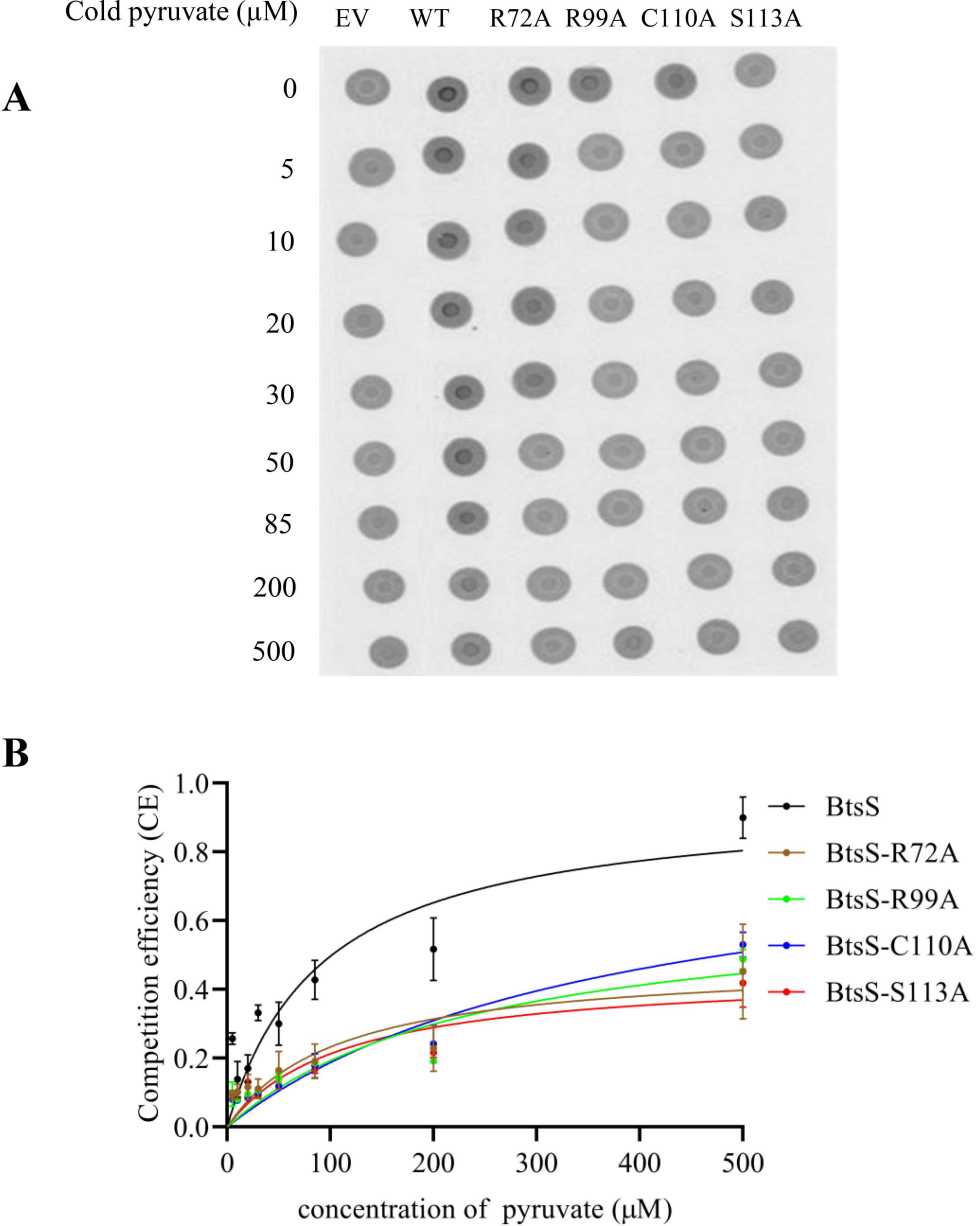
Binding of pyruvate to BtsS *in vitro*. (**A**) DRaCALA image of interactions between pyruvate and BtsS (WT) or BtsS-R72A, BtsS-R99A, BtsS-C110A, BtsS-S113A, or no BtsS (empty vector[EV]) in membrane vesicles of *E. coli* Δ*btsSR*Δ*ypdABC*. Protein–ligand (15 µM radiolabeled ^14^C-pyruvate) mixtures were spotted on a nitrocellulose membrane, allowed to dry before imaging using a PhosphoImager. (**B**) Determination of the dissociation constant (*K_d_*) for the receptor–pyruvate complex for the BtsS variants compared with wild type. Competition efficiency (CE) CE = (*F*_B(NC)_− *F*_B(pyr)_)/*F*_B(NC)_ (see Materials and Methods for details) was plotted as a function of pyruvate concentration. The CE value reached 1.0 in the presence of 50 mM cold pyruvate. The best-fit curves were determined by nonlinear regression using the equation *y* = *B*_max_*x/(*K_d_* +*x*). *K_d_* values were determined in *n* = 3 independent experiments, and the error bars represent the standard deviation of the means. *K_d_* of BtsS, BtsS-R72A, BtsS-R99A, BtsS-C110A, and BtsS-S113A is 67.3 ± 10.6 µM, 107.2 ± 12.3 µM, 245.4 ± 10.1 µM, 375.2 ± 21.2 µM, and 283.6 ± 8.0 µM, respectively.

Next, we investigated the ability of the BtsS variants with replacements at Arg72, Arg99, Cys110, and Ser113 for altered affinities to bind pyruvate *in vitro*. For this purpose, we used the differential radial capillary action of ligand assay (DRaCALA) (29), which we had previously used to quantitate the interaction between BtsS and pyruvate *in vitro* (4). In this assay, a radiolabeled ligand is used. The principle of DRaCALA is based on the immobilization of the protein-ligand complex on a nitrocellulose membrane, while the unbound ligand diffuses radially with the buffer due to the capillary action of the membrane. Thus, both total ligand and protein-bound ligand are detected. The fraction of ligand bound to protein, defined as *F*_*B*_, is calculated from the signal intensity of the area with protein (inner circle) and the signal intensity of the whole area (outer circle). We used membrane vesicles prepared from the *E. coli* ∆*btsSR*∆*ypdABC* mutant after overproduction of BtsS (WT) or BtsS-R72A, BtsS-R99A, BtsS-C110A, BtsS-S113A, and incubated equal amounts of protein with increasing concentrations of pyruvate, each containing 15 µM radiolabeled ^14^C-pyruvate. We then dropped the mixture onto a nitrocellulose membrane (Fig. 5A). Migration of the ligand by capillary action was detected after exposure of the membrane to a phosphoscreen followed by image analysis. As a negative control, we used membrane vesicles of the *E. coli* ∆*btsSR*∆*ypdABC* mutant, in which no pyruvate-binding proteins should be found (EV) (Fig. 5A). Dark inner circles that were not seen in the negative control were detected for the wild type, indicating binding of ^14^C-pyruvate and resulting in an *F*_*B*_ value of 0.198. To determine pyruvate affinity, the total pyruvate concentration was steadily increased by adding cold pyruvate, which resulted in less pronounced dark rings due to competition between cold and radiolabeled pyruvate. Background (EV) was subtracted for all concentrations and *K_d_* values were calculated. For wild-type BtsS, a *K_d_* value of 67.3 ± 10.6 µM was determined, consistent with previous measurements (4). For the BtsS variants with amino acid substitutions that affected pyruvate sensing, the following *K_d_* values were determined: BtsS-R72A *K_d_* = 107.2 ± 12.3 µM; BtsS-R99A *K_d_* = 245.4 ± 10.1 µM; BtsS-C110A *K_d_* = 375.2 ± 21.2 µM; BtsS-S113A *K_d_* = 283.6 ± 8.0 µM (Fig. 5B). These results indicate that the individual replacement of Arg72, Arg99, Cys110, and Ser113 with alanine decreases the affinity of BtsS for pyruvate, suggesting that these amino acids may be part of the pyruvate-binding site.

### Model of the 3D-structure of BtsS and the pyruvate-binding site

**Fig 6:**
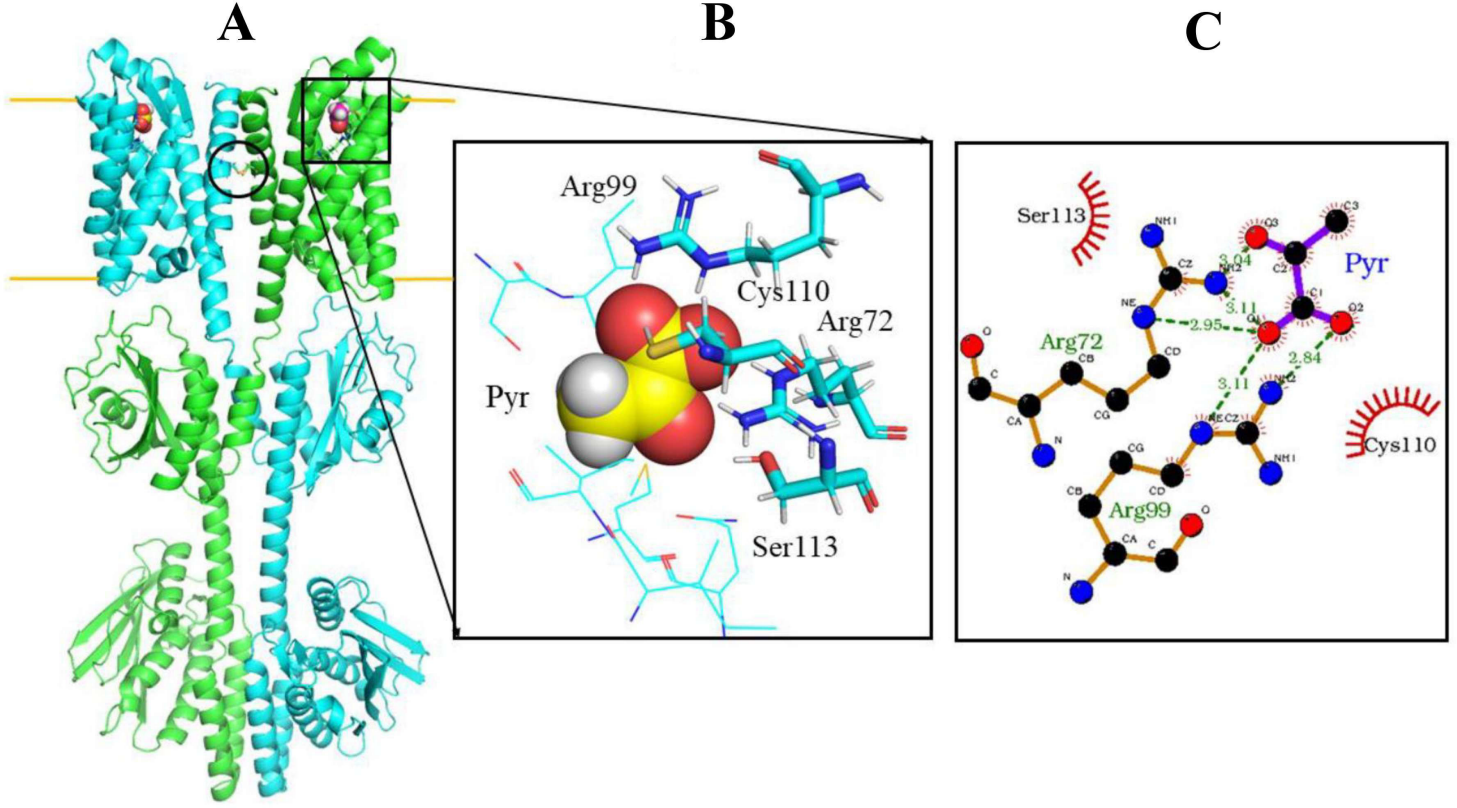
(**A**) AlphaFold2-based structural model of the BtsS dimer with bound pyruvate. Each BtsS monomer is represented as a green or light blue cartoon and the membrane boundaries are indicated by yellow lines. The position of a putative disulfide bridge between the monomers is encircled. (**B**) The putative pyruvate (Pyr) binding geometry (pyruvate in van der Waals representation) is shown enlarged in the right panel, including the arrangement of residues important for binding as stick model. (**C**) Schematic 2D contact view of pyruvate (Pyr) binding to BtsS. Hydrogen bonds between Arg72/99 and pyruvate as well as direct contacts to Cys110 are indicated. Ser113 contacts and stabilizes the Arg72 placement.

Based on the experimental information, we assume that pyruvate binds near the residues identified by mutagenesis. From structural modeling and MD-based positioning of the pyruvate in the N-terminal domain (Fig. 6), we found that Cys110 is in close contact with the pyruvate (but does not form hydrogen bonds), whereas the two Arg residues form stable hydrogen bonds with the ligand. The pyruvate-binding geometry is stable during MD simulation (Fig. S3). The guanidinium group of Arg99 forms direct hydrogen bonds with the two oxygen atoms of the pyruvate carboxyl group, and the carbonyl oxygen in pyruvate forms a bifurcated hydrogen bond with the guanidinium group of Arg72. Interestingly, although Ser113 is also located close to pyruvate, no direct hydrogen bond is formed, but a close H-bond contact to Arg72 is observed in the model structure. Therefore, Ser113 might play a decisive role in placing and stabilizing the position of the guanidinium group of Arg72, allowing close contact with pyruvate. Since pyruvate is negatively charged, the positively charged Arg72 and Arg99 provide strong and favorable electrostatic interactions to stabilize the complex. We would like to emphasize that we only provide a structural model and how pyruvate can be bound. This model may contain errors and is not suitable for quantitative analysis of residue contributions to binding.

### Autokinase activity and dimerization of BtsS

Upon perception of a stimulus, histidine kinases generally transduce the information into an intracellular signal by initiating a phosphorylation cascade (30). In our previous studies, we did not detect autophosphorylation activity of BtsS (4). This was not surprising at first, because BtsS belongs to the LytS-type histidine kinases, which have several unusual features (31): in the H-box, the conserved histidine is preceded by a proline, and followed by a phenylalanine instead of the usual acidic residue. The N-box has an unusual signature sequence, the F-box is not conserved, and the distance between the H- and X-box is reduced.

**Fig 7:**
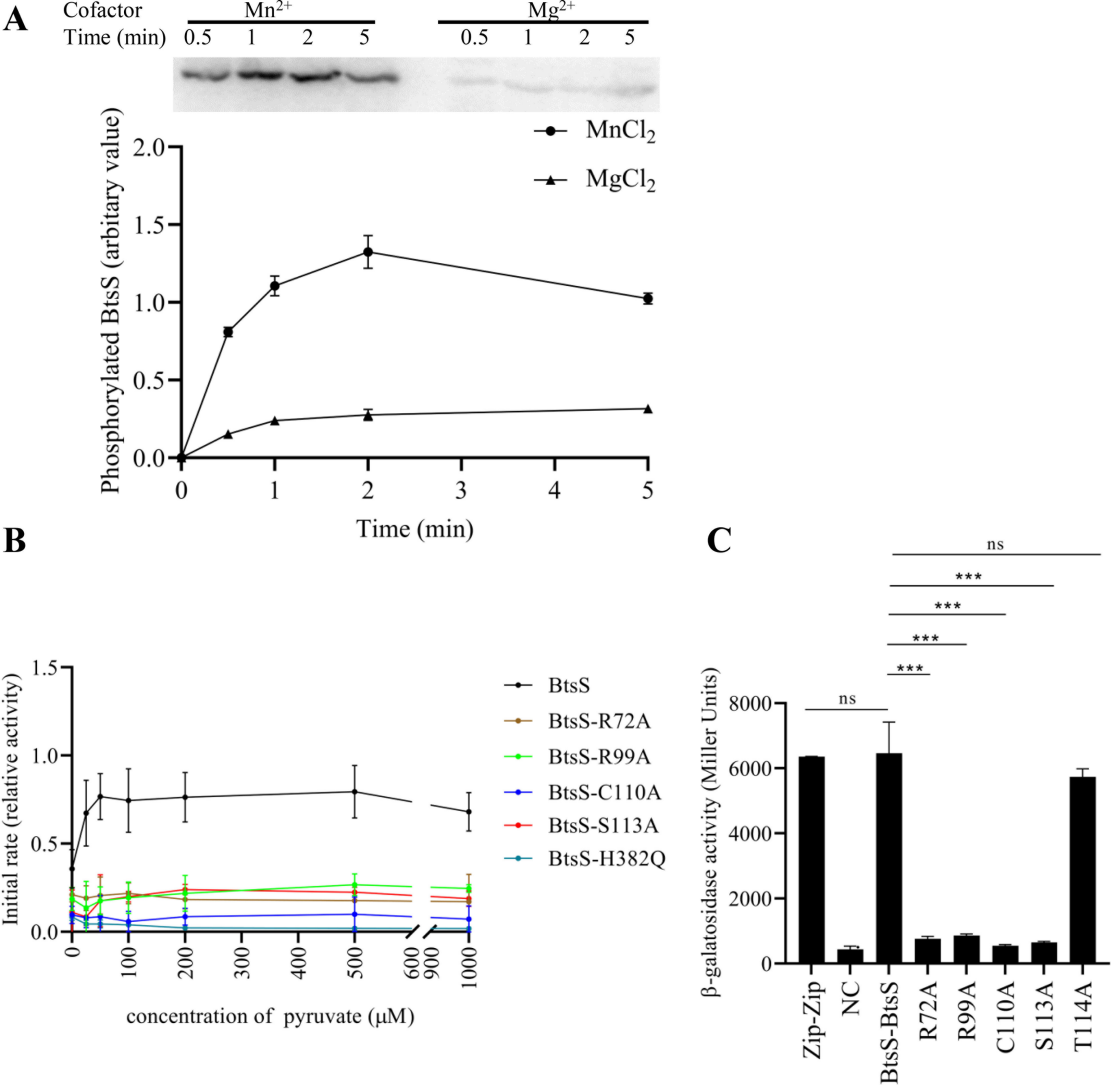
Autophosphorylation activity and dimerization of BtsS and its variants. (**A**) Time-dependent autophosphorylation of BtsS in the presence of Mg^2+^ and Mn^2+^. Membrane vesicles from *E. coli* TKR2000 producing BtsS-6His were incubated in the presence of 50 µM pyruvate. At time zero, 20 µM [γ-^32^P]ATP (2.38 Ci/mmol) and either 5 mM MgCl_2_ or MnCl_2_ was added. Reactions were stopped at the indicated time point, and phosphorylated proteins were separated by SDS-PAGE, followed by phosphoimage analysis. Upper panel: phosphoimages of representative gels, lower panel: quantified values of radiolabeled BtsS using ImageQuant. Shown are the relative values normalized to the phosphorylation level of wild-type BtsS after incubation with 50 µM pyruvate and 5 mM MnCl_2_ for 5 min (value of 1.0). (**B**) The effect of increasing pyruvate concentrations on the initial rate of autophosphorylation of BtsS, BtsS-R72A, BtsS-R99A, BtsS-C110A, BtsS-S113A, and BtsS-H382Q. Samples were processed and data were calculated as described in A (see also Fig. S3 to S6). (**C**) BACTH-based reporter assay to detect the dimerization of BtsS and its variants. *E. coli* BTH101 was co-transformed with plasmid pairs encoding the C-terminal T18 and C-terminal T25 hybrids. Cells were grown in 0.1× LB medium supplemented with 0.5 mM IPTG and 100 µM pyruvate at 37°C overnight. The activity of the reporter enzyme β-galactosidase was determined and served as a measure of the strength of the interaction. Dimerization of yeast leucine zipper protein GCN4 (Zip-Zip) was used as a positive control. The pUT18 and pKT25N vectors served as negative control (NC). All experiments were performed in triplicate, and error bars indicate standard deviation of the means. Statistics: Student’s unpaired two-sided *t*-test (***P < 0.001; **P < 0.01; *P < 0.05; ns *P* > 0.05).

In this study, we again tested the autophosphorylation activity of BtsS and found that the cofactor makes a difference. When BtsS was phosphorylated in the presence of Mg^2+^-ATP, only very weak autophosphorylation was detectable (Fig. 7A). The autophosphorylation activity of BtsS was about 10-fold higher when Mn^2+^-ATP was used (Fig. 7A). The predicted phosphorylation site at His382 was confirmed, as the variant BtsS-H382Q could not be phosphorylated (Fig. 7B).

We then asked whether pyruvate binding stimulates autophosphorylation activity. To test this, the autokinase activity of wild-type BtsS and all pyruvate-insensitive BtsS variants was determined in the presence of different pyruvate concentrations. For wild type, we found a 1.6-fold increase of the initial rate in the presence of 25 µM pyruvate. With 50 and 100 µM pyruvate, the initial rate was also increased (Fig. 7B; Fig. S4). The presence of even higher concentrations of pyruvate (200, 500, 1,000 µM) did not further stimulate the initial rates (Fig. 7B; Fig. S4). In contrast, all pyruvate-insensitive BtsS variants, namely BtsS-R72A, BtsS-R99A, BtsS-C110A, BtsS-S113A, were characterized by lower, and pyruvate-independent autophosphorylation activity (Fig. 7B; Fig. S4 to S7). Initial rates were determined to be 2.3- to 3.6-fold lower compared to wild-type BtsS. The decreased autokinase activities of the variants could also be explained by changes in dimerization (see below). The observed effects were not due to the amino acid exchanges *per se*, as BtsS-T114A (Fig. S8) and BtsS-R192A (Fig. S9) retained the concentration-dependent response to pyruvate.

In addition to measuring the autophosphorylation activity, we also determined the dimerization of BtsS and its variants *in vivo*. Dimerization is a prerequisite for all histidine kinases to mediate autophosphorylation by ATP either *in trans* or *in cis* (32). To test dimerization, we used the bacterial adenylate cyclase-based two-hybrid system (33). As previously observed (14), wild-type BtsS forms a dimer in the presence of pyruvate, as indicated by a high β-galactosidase activity that was in the same range as the positive control (leucine zipper protein GCN4) (Fig. 7C). In contrast, individual replacement of each of the amino acids involved in pyruvate binding prevented dimerization of BtsS-R72A, BtsS-R99A, BtsS-C110A, and BtsS-S113A, as indicated by very low β-galactosidase activities that were almost in the same range as the value of the negative control. However, these variants were produced as intact hybrid proteins as confirmed by SDS-PAGE and immunodetection (Fig. S10). As control, we tested dimerization of the variant BtsS-T114A, which behaved like the wild type (Fig. 7C).

## DISCUSSION

In general, histidine kinases have an even number of transmembrane helices and a cytoplasmic location of the N-terminus. There are exceptions, such as the histidine kinase AgrC of *Staphylococcus aureus* (34) and the hybrid histidine kinase LuxN of *Vibrio harveyi* (24, 35), in which the N-terminus is located outside the cytoplasm and which contain an odd number of transmembrane helices. The BtsS/BtsR histidine kinase system belongs to the LytS/LytTR-like family. All LytS-like histidine kinases have the 5TMR-LYT (for 5 transmembrane receptors of the LytS-YhcK type) input domain that receives signals (36). This domain, with an average of 169 amino acids, is present in about 88 different protein architectures and predominantly found in histidine kinases, but also occurs in combination with c-di-GMP synthetases (GGDEF domain-containing proteins) (36). Bioinformatics hydrophobicity analyses (using TMHMM, PsiPred, and Protscale) suggested that BtsS is anchored in the membrane with at least six transmembrane helices, consistent with the fact that the first 36 amino acids do not belong to the 5TMR-LYT domain (37 – 39) (Fig. S1A). Based on the 3D-structure prediction with AlphaFold2, an organization of the input domain with seven transmembrane helices was proposed (Fig. 1 and 6). We therefore experimentally tested the location of the N-terminus of BtsS using the maltose-binding protein as a reporter. *malE* was fused to the 5′-end of *btsS,* and only a hybrid protein in which MalE was localized to the periplasmic side was able to complement a ∆*malE* mutant (indicator: growth with maltose as the sole C source) and functioned as a pyruvate sensor (Fig. 2). These results support a periplasmic location of the N-terminus of BtsS and imply that this receptor is anchored in the cytoplasmic membrane with seven transmembrane domains (Fig. 2 and 6). Seven-transmembrane receptors constitute the largest, most ubiquitous, and most versatile family of membrane receptors in eukaryotes, including rhodopsin, G-protein coupled receptors (40). Comparative genomic analysis also identified several families of seven-transmembrane receptors, in particular the 7TMR-DISMED1, 7TMR-DISMED2, and 7TMR-HD families in bacteria. To our knowledge, the results presented here are the first example of a seven-transmembrane receptor belonging to the LytS-like histidine kinase family.

Some structures of crystallized pyruvate-binding proteins provide clues to the amino acids involved in formation of the binding pocket. In the sensor domain of the histidine kinase KinD from *Bacillus subtilis*, which surprisingly contained pyruvate, two arginines and the hydroxyl group of serine are in the contact with the carboxyl group of pyruvate. The keto group makes contacts with serine and tyrosine via a water molecule (PDB: 4JGP) (26). In the pyruvate formate-lyase from *Escherichia coli* (PDB: 1MZO) (25), pyruvate is located in a cleft close to Cys418 and Cys419, with the carboxyl group in contact with Arg176 and Arg435 and the methyl group within van der Waals distance of Phe327. Arg548 and Gln552 of the pyruvate carboxylase from *Rhizobium etli* allow the binding of pyruvate (PDB: 4JX4, 4JX5, 4JX6) (41, 42). For the high-affinity pyruvate receptor BtsS, we identified Arg72, Arg99, Cys110, and Ser113 as amino acids involved in pyruvate binding. Based on the modeling of the 3D structure and MD-based positioning of the pyruvate, the two Arg residues form stable hydrogen bonds with the ligand (Fig. 6). Cys110 is in close contact with pyruvate. Although in the model Ser113 is also located in the vicinity of pyruvate, no direct hydrogen bond is formed, but a close H-bond contact with Arg72 is observed in the structure. Therefore, Ser113 may play a crucial role in positioning the guanidinium group of Arg72 so that close contact with pyruvate is possible. Pyruvate does not bind to amino acids exposed to membrane lipids in our model, but into a partially buried pocket. The residues that form this pocket are partially exposed to the solvent. Whether the individual replacement of these four amino acids affects the folding of the receptor could not be answered by the modeling approach. However, the fact that all of these variants were inserted into the cytosolic membrane and were still enzymatically active *in vitro* argues against unfolding.

The four amino acids identified in this work are essential for BtsS, since replacement of even one of these amino acids reduces or completely prevents induction of *btsT in vivo* (Fig. 4). BtsS is a high-affinity receptor for pyruvate. For the wild-type receptor, we determined a *K_d_* value of 67.3 ± 10.6 µM, which is in the same range as our previous measurement (58.6 µM) (4). The *K_d_* values measured for the variants BtsS-R72A, BtsS-R99A, BtsS-C110A, and BtsS-S113A are higher, indicating that their affinity for pyruvate is lower than that of the wild type. However, these changes are relatively small and cannot be the sole reason why the variants fail to induce *btsT* even at higher extracellular pyruvate concentrations. Rather, the lack of pyruvate-stimulable phosphorylation and/or dimerization of these BtsS variants prevent signal transduction (Fig. 7). ClustalW was utilized to perform a multiple sequence alignment of BtsS and various LytS histidine kinases (43) (Fig. S11). Sequence comparison revealed that both arginines (R72 and R99 in BtsS) are highly conserved. In contrast, Cys110 and Ser113 (numbering of BtsS) are more variable and are only 71.4% and 78.6% conserved, respectively (Fig. S11). In some receptors, Cys110 is replaced by Tyr, and Ser113 by Ala or Thr (Fig. S11). LytS-type kinases with changes at the corresponding position of Cys and Ser might have a different substrate specificity, e.g., for lactate, but this has to be tested experimentally first.

Until recently, we and others (44) could not demonstrate autophosphorylation activity for BtsS. In this study, we found that BtsS requires Mn^2+^-ATP instead of Mg^2+^-ATP for *in vitro* autokinase activity, which is unusual for histidine kinases. There are some histidine kinases from plants and some serine/tyrosine kinases that prefer Mn^2+^ for their activity (45, 46). In bacteria, only the hybrid histidine kinase FrzE from *Myxococcus xanthus* is known to autophosphorylate in the presence of Mn^2+^ (47). Mn^2+^ is required as a free ion or a cofactor of superoxide dismutase for resistance to oxidative stress (48). Pyruvate is a known scavenger of reactive oxygen radicals (49, 50). Nevertheless, a link between BtsS/BtsR activation or pyruvate uptake and oxidative stress is currently unclear and needs further experimentation. It should be noted that Cys15 in BtsS may be able to form a disulfide bridge between two dimers (Fig. 6).

We also found that the autokinase activity of BtsS is stimulated in the presence of pyruvate and that there is linear correlation between the perceived pyruvate and the increase in activity in the concentration range from 0 to 100 µM pyruvate. At concentrations greater than 100 µM pyruvate, there is no further increase in activity (Fig. 7). This concentration range for stimulation of autokinase activity fits very well with the observed affinity of wild-type BtsS for binding pyruvate.

This study provides comprehensive insights into the molecular mechanism of stimulus-dependent activation of BtsS, the first member of the LytS histidine kinases. When Arg72, Arg99, Cys110, and Ser113, localized in three different transmembrane domains of the 7-TM receptor, bind pyruvate, this leads to Mn^2+^-ATP-dependent autophosphorylation at the conserved His382. The corresponding amino acids are located at conformation-dependent positions, as the exchange of individual amino acids also affects the dimerization of BtsS. The elucidation of the pyruvate-binding site of BtsS now also opens up the possibility of redesigning this sensor for pyruvate-like ligands such as lactate, which can be used as bio-sensor in biomedicine.

## MATERIALS AND METHODS

### Strains and plasmids

The bacterial strains and plasmids used in this study are listed in Table 1. *E. coli* DH5α was used for plasmid construction, *E. coli* MG1655 Δ*btsSR* for *in vivo btsT* expression assays, *E. coli* MG1655 Δ*btsSR*Δ*ypdABC* for *in vitro* protein-ligand binding assays, *E. coli* TKR2000 for autophosphorylation assays, *E. coli* BTH101 for dimerization assays, and *E. coli* MM39 for BtsS localization assays.

**TABLE 1:** Bacterial strains and plasmids used in this study

Strain or plasmid	Genotype or description	Reference
*E. coli* strains		
DH5α	F⁻ λ⁻ *endA1 glnV44 thi-1 recA1 relA1 gyrA96 deoR nupG* ф80d *lac* ΔM15 Δ(*lacZYA-argF*) U169 *hsdR17*(r_K_^⁻^ m_K_^+^)	(51)
MG1655 ΔbtsSR	F⁻ λ⁻ i*lvG rfb50 rph-1* Δ*btsSR*	(5)
MG1655 ΔbtsSRΔypdABC	F⁻ λ⁻ *ilvG rfb50 rph-1* Δ*btsSR*Δ*ypdABC*	(18)
BTH101	F⁻ *cyaA-99 araD139 galE15 galK16 rpsL1 hsdR2 mcrA1 mcrB1*	(33)
TKR2000	Δ*kdpFABCDE trkA405 trkD1 atp706 nagA thi rha lacZ*	(52)
MM39	*araD lacI* Δ*U1269 malE444*, Str^r^	(24)
Plasmids		
pBBR-btsT-lux	P_*btsT*_-212/+88 cloned in the BamHI and EcoRI sites of pBBR1-MCS5-TT-RBS-lux; Gm^r^	(5)
pBAD24	Arabinose-inducible P_*BAD*_ promoter, pBR322 ori; Amp^r^	(53)
pBAD24-btsS-6His	*btsS-6His* cloned into the EcoRI and XbaI sites of pBAD24; Amp^r^	(5)
pBAD24-btsS-R72A-6His	Replacement of R72A in pBAD24-btsS-6His; Amp^r^	This work
pBAD24-btsS-R99A-6His	Replacement of R99A in pBAD24-btsS-6His; Amp^r^	This work
pBAD24-btsS-C110A-6His	Replacement of C110A in pBAD24-btsS-6His; Amp^r^	This work
pBAD24-btsS-S113A-6His	Replacement of S113A in pBAD24-btsS-6His; Amp^r^	This work
pBAD24-btsS-H382Q-6His	Replacement of H382Q in pBAD24-btsS-6His; Amp^r^	(5)
pBAD24-btsS-T114A-6His	Replacement of T114A in pBAD24-btsS-6His; Amp^r^	This work
pBAD24-btsS-R192A-6His	Replacement of R192A in pBAD24-btsS-6His; Amp^r^	This work
pBAD24-btsS/R	*btsS-Flag/btsR-6His* cloned into the EcoRI and XbaI sites of pBAD24; Amp^r^	This work
pBAD24-btsS-K26A/btsR	Replacement of K26A in pBAD24-btsS/R; Amp^r^	This work
pBAD24-btsS-K26R/btsR	Replacement of K26R in pBAD24-btsS/R; Amp^r^	This work
pBAD24-btsS-K26H/btsR	Replacement of K26H in pBAD24-btsS/R; Amp^r^	This work
pBAD24-btsS-K26E/btsR	Replacement of K26E in pBAD24-btsS/R; Amp^r^	This work
pBAD24-btsS-R39A/btsR	Replacement of R39A in pBAD24-btsS/R; Amp^r^	This work
pBAD24-btsS-R39K/btsR	Replacement of R39K in pBAD24-btsS/R; Amp^r^	This work
pBAD24-btsS-R39H/btsR	Replacement of R39H in pBAD24-btsS/R; Amp^r^	This work
pBAD24-btsS-K43A/btsR	Replacement of K43A in pBAD24-btsS/R; Amp^r^	This work
pBAD24-btsS-R72A/btsR	Replacement of R72A in pBAD24-btsS/R; Amp^r^	This work
pBAD24-btsS-R72E/btsR	Replacement of R72E in pBAD24-btsS/R; Amp^r^	This work
pBAD24-btsS-R72H/btsR	Replacement of R72H in pBAD24-btsS/R; Amp^r^	This work
pBAD24-btsS-R72K/btsR	Replacement of R72K in pBAD24-btsS/R; Amp^r^	This work
pBAD24-btsS-R72Q/btsR	Replacement of R72Q in pBAD24-btsS/R; Amp^r^	This work
pBAD24-btsS-R99A/btsR	Replacement of R99A in pBAD24-btsS/R; Amp^r^	This work
pBAD24-btsS-R99E/btsR	Replacement of R99E in pBAD24-btsS/R; Amp^r^	This work
pBAD24-btsS-R99Q/btsR	Replacement of R99Q in pBAD24-btsS/R; Amp^r^	This work
pBAD24-btsS-R99H/btsR	Replacement of R99H in pBAD24-btsS/R; Amp^r^	This work
pBAD24-btsS-R99K/btsR	Replacement of R99K in pBAD24-btsS/R; Amp^r^	This work
pBAD24-btsS-C110A/btsR	Replacement of C110A in pBAD24-btsS/R; Amp^r^	This work
pBAD24-btsS-C110V/btsR	Replacement of C110V in pBAD24-btsS/R; Amp^r^	This work
pBAD24-btsS-C110G/btsR	Replacement of C110G in pBAD24-btsS/R; Amp^r^	This work
pBAD24-btsS-C110S/btsR	Replacement of C110S in pBAD24-btsS/R; Amp^r^	This work
pBAD24-btsS-S113A/btsR	Replacement of S113A in pBAD24-btsS/R; Amp^r^	This work
pBAD24-btsS-S113T/btsR	Replacement of S113T in pBAD24-btsS/R; Amp^r^	This work
pBAD24-btsS-S113V/btsR	Replacement of S113V in pBAD24-btsS/R; Amp^r^	This work
pBAD24-btsS-S113G/btsR	Replacement of S113G in pBAD24-btsS/R; Amp^r^	This work
pBAD24-btsS-T114A/btsR	Replacement of T114A in pBAD24-btsS/R; Amp^r^	This work
pBAD24-btsS-T114V/btsR	Replacement of T114V in pBAD24-btsS/R; Amp^r^	This work
pBAD24-btsS-T114G/btsR	Replacement of T114G in pBAD24-btsS/R; Amp^r^	This work
pBAD24-btsS-T114S/btsR	Replacement of T114S in pBAD24-btsS/R; Amp^r^	This work
pBAD24-btsS-R130A/btsR	Replacement of R130A in pBAD24-btsS/R; Amp^r^	This work
pBAD24-btsS-R131A/btsR	Replacement of R131A in pBAD24-btsS/R; Amp^r^	This work
pBAD24-btsS-R131E/btsR	Replacement of R131E in pBAD24-btsS/R; Amp^r^	This work
pBAD24-btsS-R131Q/btsR	Replacement of R131Q in pBAD24-btsS/R; Amp^r^	This work
pBAD24-btsS-R133A/btsR	Replacement of R133A in pBAD24-btsS/R; Amp^r^	This work
pBAD24-btsS-K136A/btsR	Replacement of K136A in pBAD24-btsS/R; Amp^r^	This work
pBAD24-btsS-R163A/btsR	Replacement of R163A in pBAD24-btsS/R; Amp^r^	This work
pBAD24-btsS-R170A/btsR	Replacement of R170A in pBAD24-btsS/R; Amp^r^	This work
pBAD24-btsS-R192A/btsR	Replacement of R192A in pBAD24-btsS/R; Amp^r^	This work
pBAD24-btsS-R192H/btsR	Replacement of R192H in pBAD24-btsS/R; Amp^r^	This work
pBAD24-btsS-R192K/btsR	Replacement of R192K in pBAD24-btsS/R; Amp^r^	This work
pBAD24-btsS-R192Q/btsR	Replacement of R192Q in pBAD24-btsS/R; Amp^r^	This work
pBAD24-btsS-R197A/btsR	Replacement of R197A in pBAD24-btsS/R; Amp^r^	This work
pBAD24-btsS-R198A/btsR	Replacement of R198A in pBAD24-btsS/R; Amp^r^	This work
pBAD24-btsS-N70A/btsR	Replacement of N70A in pBAD24-btsS/R; Amp^r^	This work
pBAD24-btsS-T71A/btsR	Replacement of T71A in pBAD24-btsS/R; Amp^r^	This work
pBAD24-btsS-T94A/btsR	Replacement of T94A in pBAD24-btsS/R; Amp^r^	This work
pBAD24-btsS-Y100A/btsR	Replacement of Y100A in pBAD24-btsS/R; Amp^r^	This work
pBAD24-btsS-S101A/btsR	Replacement of S101A in pBAD24-btsS/R; Amp^r^	This work
pBAD24-btsS-T106A/btsR	Replacement of T106A in pBAD24-btsS/R; Amp^r^	This work
pKT25N	Vector of the bacterial two-hybrid system. Carries T25 fragment (720 bp) of *сyaA*, Kan^r^	(33)
pUT18	Vector of the bacterial two-hybrid system. Carries T18 fragment ((616 bp) of *cyaA*, Amp^r^	(33)
pUT18-btsS-6His	*btsS-6His* cloned in XbaI and BamHI sites of pUT18 resulting in C-terminal CyaA-T18-protein hybrid, Kan^r^	This work
pKT25N-btsS-6His	*btsS-6His* cloned in XbaI and BamHI sites of pKT25N resulting in C-terminal CyaA-T25-protein hybrid, Amp^r^	This work
pUT18-zip	The leucine zipper GCN4 gene is fused in frame to the T18 fragment, Amp^r^	(33)
pKT25N-zip	The leucine zipper GCN4 gene is fused in frame to the T25 fragment, Kan^r^	(33)
pUT18-btsS-R72A-6His	Replacement of R72A in pUT18-btsS-6His; Amp^r^	This work
pUT18-btsS-R99A-6His	Replacement of R99A in pUT18-btsS-6His; Amp^r^	This work
pUT18-btsS-C110A-6His	Replacement of C110A in pUT18-btsS-6His; Amp^r^	This work
pUT18-btsS-S113A-6His	Replacement of S113A in pUT18-btsS-6His; Amp^r^	This work
pUT18-btsS-T114A-6His	Replacement of T114A in pUT18-btsS-6His; Amp^r^	This work
pKT25N-btsS-R72A-6His	Replacement of R72A in pKT25N-btsS-6His; Kan^r^	This work
pKT25N-btsS-R99A-6His	Replacement of R99A in pKT25N-btsS-6His; Kan^r^	This work
pKT25N-btsS-C110A-6His	Replacement of C110A in pKT25N-btsS-6His; Kan^r^	This work
pKT25N-btsS-S113A-6His	Replacement of S113A in pKT25N-btsS-6His; Kan^r^	This work
pKT25N-btsS-T114A-6His	Replacement of T114A in pKT25N-btsS-6His; Kan^r^	This work
pMAL-p2X	Expression vector encoding MBP, Amp^r^	New England Biolabs
pMAL-c2X	Expression vector, encoding MBP without signal peptide, Amp^r^	New England Biolabs
pMALp-btsS	*btsS* cloned in BamHI and HindIII sites of pMAL-p2X encoding a hybrid protein of MalEp and BtsS, Amp^r^	This work
pMALc-btsS	*btsS* cloned in BamHI and HindIII sites of pMAL-c2X encoding a hybrid protein of MalEc and BtsS, Amp^r^	This work
pBAD24-pMALp-btsS/R	*malEp-btsS/R* cloned into the EcoRI and XbaI sites of pBAD24; Amp^r^	This work
pBAD24-pMALc-btsS/R	*malEc-btsS/R* cloned into the EcoRI and XbaI sites of pBAD24; Amp^r^	This work

### Construction of *btsS* variants

All *btsS* variants were constructed using the Q5 site-directed mutagenesis kit (New England BioLabs). Plasmid DNAs were isolated using a HiYield plasmid mini kit (Sued Laborbedarf). DNA fragments were purified from agarose gels using a HiYield PCR cleanup and gel extraction kit (Sued Laborbedarf). Restriction enzymes and other DNA-modifying enzymes were purchased from New England BioLabs and used according to the manufacturer’s directions.

### Use of MalE as reporter protein for the N-terminus

*E. coli* MM39 was transformed with plasmid pMAL-p2X, pMAL-c2X, pMALp-*btsS,* or pMALc-*btsS*, and then plated on LB agar plates with streptomycin (50 µg/mL) and ampicillin (100 µg/mL). In parallel, *E. coli* MG1655 and MM39 were streaked on LB agar plates as controls. Subsequently, individual colonies were re-streaked on M9 minimal medium agar plates supplemented with 0.1% (wt/vol) maltose as sole C source and incubated at 37°C for 36 h (24).

The *in vivo* activity of BtsS/R was measured by monitoring the expression of *btsT* (4). *E. coli* MG1655Δ*btsSR* was co-transformed with plasmids pBBR-*btsT-lux* and pBAD24 (negative control) or pBAD24-*btsS/R* (positive control) or pBAD24-pMALp-*btsS/R* or pBAD24-pMALc-*btsS/R*. Overnight cultures in LB medium were used to inoculate M9 minimal medium containing 5 mM pyruvate, L-lactate, or succinate, respectively, supplementary with 15 mM succinate and incubated in a plate reader (BMG Labtech CLARIOstar, Germany) at 37°C. Both OD_600_ and luminescence were determined continuously. Luminescence was determined as relative light units (RLU counts s^−1^) related to the OD_600_.

### *In vivo* signal transduction assay

*E. coli* MG1655Δ*btsSR* was co-transformed with plasmids pBAD24-*btsS*/*R* and pBBR-*btsT-lux* or pBAD24-*btsS/R* variants and pBBR-*btsT-lux*. After overnight incubation, a colony was inoculated in 5 mL LB medium with the appropriate antibiotics (ampicillin and gentamycin) and cells were grown at 37°C until OD_600_ reached 0.5. Cells were transferred to M9 minimal medium supplemented with 5 mM pyruvate or lactate or succinate (always plus 15 mM succinate to obtain a total C concentration of 20 mM) to give a starting optical density at 600 nm (OD_600_) of 0.05 and incubated at 37°C with constant agitation. Growth (OD_600_) and luminescence were measured at 10 min intervals in a luminescence reader (BMG Labtech CLARIOstar, Germany). Using the same protocol, different concentrations of pyruvate (5 mM, 10 mM, 20 mM, and 50 mM) were tested as the C source, with the total C concentration kept constant at 50 mM in each case by addition of succinate. Alternatively, overnight grown cells were inoculated in 0.1× LB medium and after 1 h different concentrations of pyruvate were added (4). Luminescence values were determined for each growth condition and were expressed as relative light units (RLU counts s^−1^) per OD_600_. Flag-tagged BtsS or variants were detected by western blot analysis using an anti-Flag antibody (Thermo Fisher) and a secondary anti-mouse antibody conjugated to alkaline phosphatase (Thermo Fisher).

### Autokinase assay

*E. coli* TKR2000 transformed with plasmid pBAD24-*btsS*-6His or variants was cultivated in KML medium (1% [wt/vol] KCl, 1% [wt/vol] tryptone and 0.5% [wt/vol] yeast extract) supplemented with ampicillin (100 µg/mL). At an OD_600_ of 0.5, 0.2% (wt/vol) arabinose was added, and cells were grown at 18°C overnight. Cells were harvested by low-speed centrifugation, and membrane vesicles were prepared as described (30). Membrane vesicles (2 mg protein/mL) containing BtsS or variants were incubated in phosphorylation buffer (25 mM Tris/HCl, pH 7.5, 5% [vol/vol] glycerol, 0.25 M NaCl, without and with pyruvate 25 µM, 50 µM, 100 µM, 200 µM, 500 µM or 1 mM, 5 mM MgCl_2_ or 5 mM MnCl_2_ and 2 mM dithiothreitol) at 25°C. Phosphorylation was initiated by the addition of 20 µM [γ-^32^P]ATP (2.38 Ci/mmol; Biotrend) (54). At different times, 20 µL aliquots were removed and the reaction was stopped by mixing with 5 µL of 5-fold SDS sample buffer (55). All samples were immediately subjected to SDS-polyacrylamide gel electrophoresis (PAGE). Proteins were transferred on a nitrocellulose membrane, and phosphorylated proteins were detected by exposure of the membrane to a Storage Phosphor Screen. Phosphorylated proteins were quantified by image analysis using Image Quant software (GE Healthcare). For each gel, a standard was loaded and set to 100% (= 1), which was phosphorylated BtsS incubated in the presence of 50 µM pyruvate, 5 mM MnCl_2_ for 5 min.

### *In vivo* dimerization studies using BACTH

Dimerization of BtsS and the variants was tested *in vivo* using the bacterial adenylate cyclase two-hybrid assay (BACTH) (33). *E. coli* BTH101 was transformed with pUT18 and pKT25N derivatives. The dimerization of leucine zipper protein GCN4 was used as a positive control. Cells were grown overnight in LB medium supplemented with 0.5 mM isopropyl-β-D-thiogalactopyranoside (IPTG) at 37°C, and used to inoculate 0.1× LB medium (nutrient limitation) (14) supplemented with 0.05 mM IPTG and 100 µM pyruvate. Cells were grown until OD_600_ reached 0.3 to 0.35. Cells were harvested and β-galactosidase activity was determined and expressed in Miller units (33). BtsS hybrid proteins were detected by western blot analysis using a monoclonal anti-His antibody and a secondary anti-mouse antibody conjugated to alkaline phosphatase (Thermo Fisher).

### Differential radial capillary action of ligand assay (DRaCALA)

The differential radial capillary action of ligand assay (DRaCALA) (29) was used to quantify the binding of pyruvate to BtsS *in vitro* as previously described (4). *E. coli* MG1655 ∆*btsSR*∆*ypdABC* was transformed with plasmid pBAD24-*btsS*-6His, pBAD24-*btsS*-R72A-6His, pBAD24-*btsS*-R99A-6His, pBAD24-*btsS*-C110A-6His, pBAD24-*btsS*-S113A-6His, or pBAD24. Membrane vesicles enriched in BtsS or its variants or control vesicles (empty vector) were mixed with 15 µM radiolabeled (3-^14^C) pyruvate (55 mCi mmol^−1^, Biotrend) and incubated for 20 min at room temperature. Triplicate 5 µL aliquots were spotted onto a nitrocellulose membrane, dried, and radioactivity was visualized using a PhosphorImager. Quantification of the areas of radioactive signal intensities was performed using ImageJ. The fraction bound (*F*_*B*_) was calculated using the equation *F*_*B*_ = (*I*_inner_ − *I*_background_)/*I*_total_ (29). To determine the *K_d_* value, increasing concentrations of cold pyruvate (0 µM to 500 µM, and 50 mM for saturation) were added to the mixture. Competition efficiency (CE) was calculated as follows: CE = (*F*_*B*(NC)_ − *F*_*B*(pyr)_)/*F*_*B*(NC)_. *F*_*B*(NC)_ is calculated from the sample without cold pyruvate (NC = no competition), and *F*_*B*(pyr)_ are all values calculated for the samples containing a mixture of ^14^C-pyruvate and different concentrations of cold pyruvate. For all *F*_*B*_ values, the value of the negative control (empty vector) was subtracted. The concentration of membrane vesicles used in this experiment was 25 mg/mL, which contain approximately 7% BtsS-6His or variants.

### Structural modeling of BtsS in complex with pyruvate

A structural model of a BtsS dimer was generated using AlphaFold2 (23) and the best ranked model (uniprot ID: P0AD14) was used for further modeling and pyruvate docking. In the model, the four residues critical for binding of pyruvate, Arg72, Arg99, Cys110, and Ser113, form a pocket in the N-terminal domain of BtsS. The arrangement of residues is similar to the geometry found in the crystal structure of pyruvate formate-lyase (*E. coli*) with bound pyruvate in the PDB: 1MZO entry. This known structure was used to first manually place pyruvate in the putative-binding pocket in the BtsS model (close to the critical residues) and to derive upper bound distance restraints between pyruvate and the side chain functional groups of the four critical residues. The modeling and pyruvate docking continued by first performing an energy minimization (2,500 steps) to remove sterical overlap in the start structure using the Amber20 molecular modeling package (56). In the next step Molecular Dynamics (MD) simulations were performed employing an implicit Generalized Born solvent model (igb = 5 option in Amber) and including positional restraints on all heavy atoms (force constant: 0.05 kcal mol^−1^Å^−2^) except for the residues in close vicinity of the bound pyruvate. In addition, the simulations included the distance restraints between pyruvate and the four critical residues in the binding region. After a step-wise heating (0.5 ns), MD simulations were continued for 10 ns and resulted in a stable binding geometry (see Fig. S3) that was again energy minimized for 2,500 steps to obtain a structural model of the complex.

## SUPPLEMENTAL MATERIAL

Supplemental Material mbio.01089-23-s0001.pdf Figures S1–S11.

ASM does not own the copyrights to Supplemental Material that may be linked to, or accessed through, an article. The authors have granted ASM a non-exclusive, world-wide license to publish the Supplemental Material files. Please contact the corresponding author directly for reuse.
