# Supplementary material for: The LytS-type histidine kinase BtsS is a 7-transmembrane receptor that binds pyruvate
Source: mBio. 2023 Sep 1;14(5):e01089-23. doi: 10.1128/mbio.01089-23 (PMC10653868; doi:10.1128/mbio.01089-23)
Supplement: Supplemental Material — Figures S1–S11. [file mbio.01089-23-s0001.pdf]

Supplementary information

The LytS-type histidine kinase BtsS is a 7-transmembrane receptor that binds pyruvate

Jin Qiu[a], Ana Gasperotti[a], Nathalie Sisattana[a], Martin Zacharias[b], and Kirsten Jung[a]

[a]Faculty of Biology, Microbiology, Ludwig-Maximilians-Universität München, Martinsried, Germany

[b]Center of Functional Protein Assemblies, Technical University of Munich, 85748 Garching, Germany

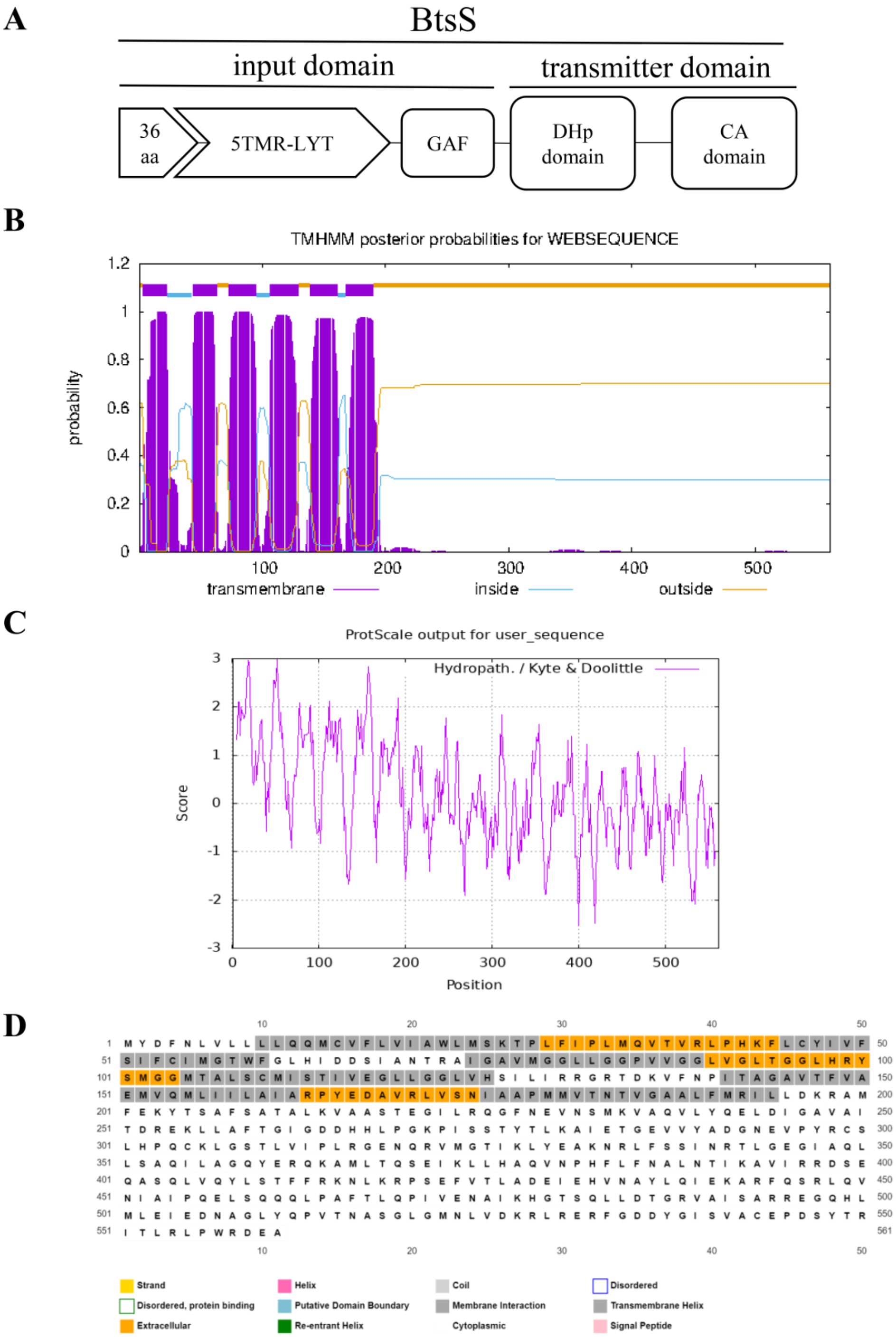

FIG S1 Domain structure of BtsS and the hydrophobicity analysis of BtsS. (A) BtsS is a histidine kinase composed of an input domain and a transmitter domain. The input domain of BtsS consists of the 5TMR-LYT domain and a GAF domain. The transmitter domain consists of a DHp domain and a CA domain. (B) Transmembrane structure prediction using TMHMM-2.0 (19, 20). (C) Hydrophilicity plot using ProtScale (21). (D) BtsS structure analysis using PsiPred (22).

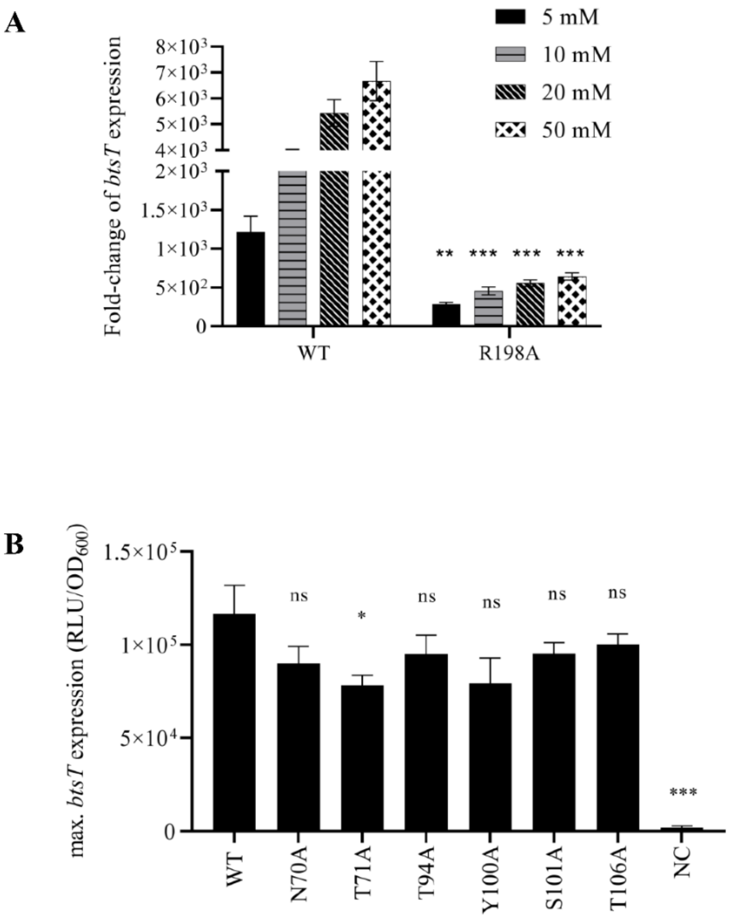

FIG S2 *In vivo btsT* expression mediated by BtsS and the indicated BtsS variants. (A) Reporter strain MG1655Δ*btsSR*/pBBR-*btsT-lux* was transformed either with pBAD24-*btsS/R* (WT) as a positive control, or pBAD24-*btsS*-R198A/*btsR*. Cells were grown with different concentrations of pyruvate (5 mM, 10 mM, 20 mM and 50 mM) as the C source, with the total C concentration kept constant at 50 mM in each case by addition of succinate, and BtsS/BtsR-mediated *btsT* expression (measured as P_{btsT}::*luxCDABE* and reported in relative light units) was measured over time. Maximum luciferase activity (RLU), normalized to an optical density (OD_{600}) of 1 are reported. Fold-change values were calculated using the succinate control. (B) The same experimental approach as in A was used, but the response to 5 mM pyruvate (supplemented with succinate for a total of 20 mM C source) was tested as described in FIG 2C. Statistics: student's unpaired two-sided t-test (*** $p < 0.001$; ** $p < 0.01$; *$p < 0.05$; ns $p > 0.05$).

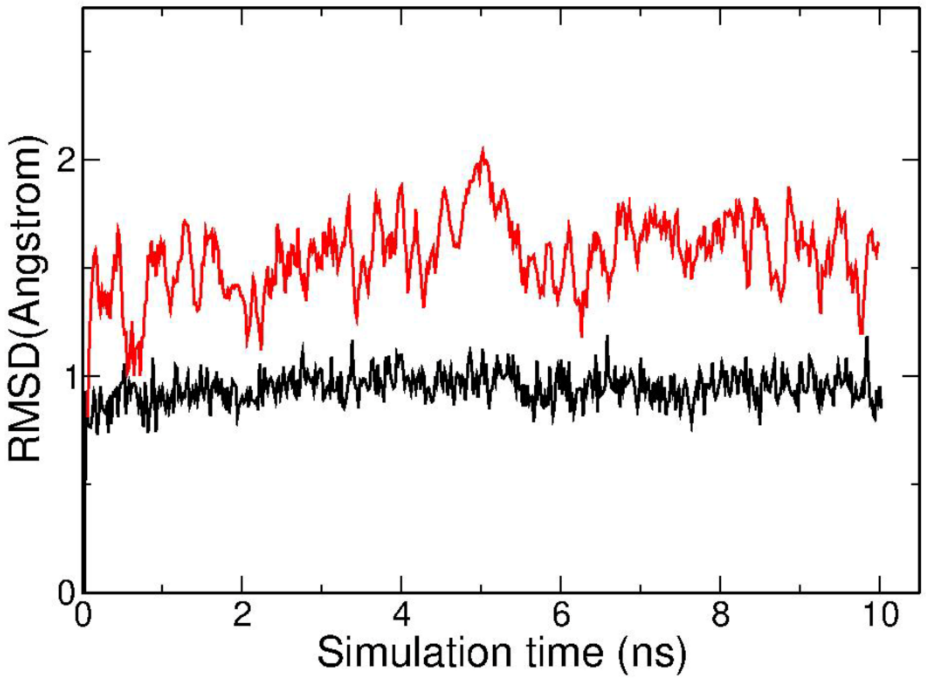

FIG S3 Root-mean-square deviation (RMSD) of protein backbone atoms (black line, pyruvate binding domain, residues 1-202) during MD simulation of the complex of BtsS with pyruvate from the starting structure. The simulation included positional restraints on the protein (force constant: 0.05 kcal $mol^{-1}Å^{-2}$). The RMSD of the pyruvate ligand (after best superposition of the pyruvate binding domain on the start structure) is also shown (red line).

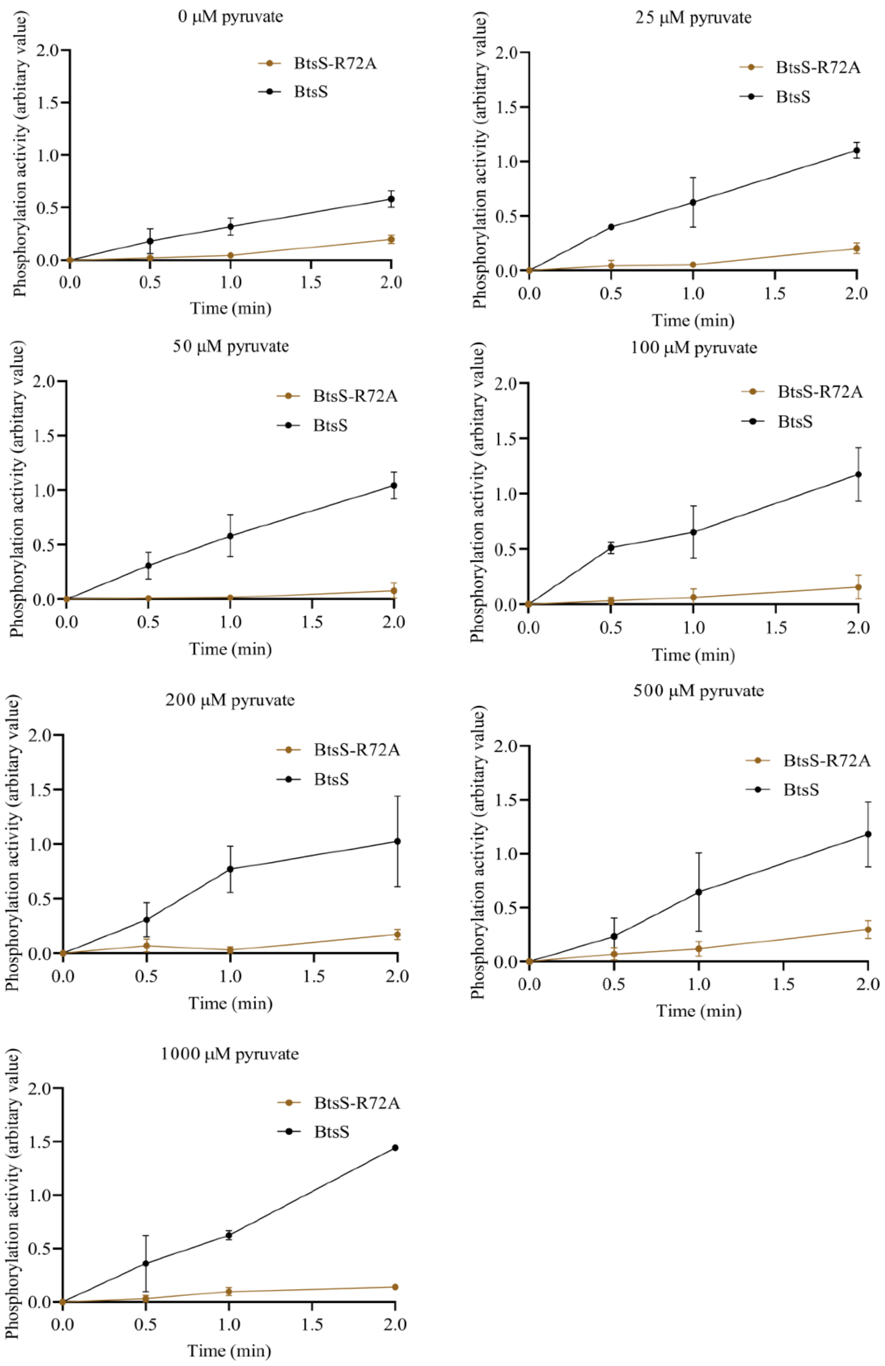

FIG S4 The influence of increasing pyruvate concentrations on the autophosphorylation activity of BtsS-R72A compared to wild-type BtsS. Membrane vesicles (2 mg/mL) were incubated in the presence of the indicated pyruvate concentrations. At time zero, 20 µM [γ-^{32}P] ATP (2.38 Ci/mmol) was added. Reactions were stopped at the indicated time points, and phosphorylated proteins were separated by SDS-PAGE, followed by phosphoimage analysis. Values are normalized to the phosphorylation level of wild-type BtsS after incubation with 50 µM pyruvate and 5 mM $MnCl_2$ for 5 min (value of 1.0).

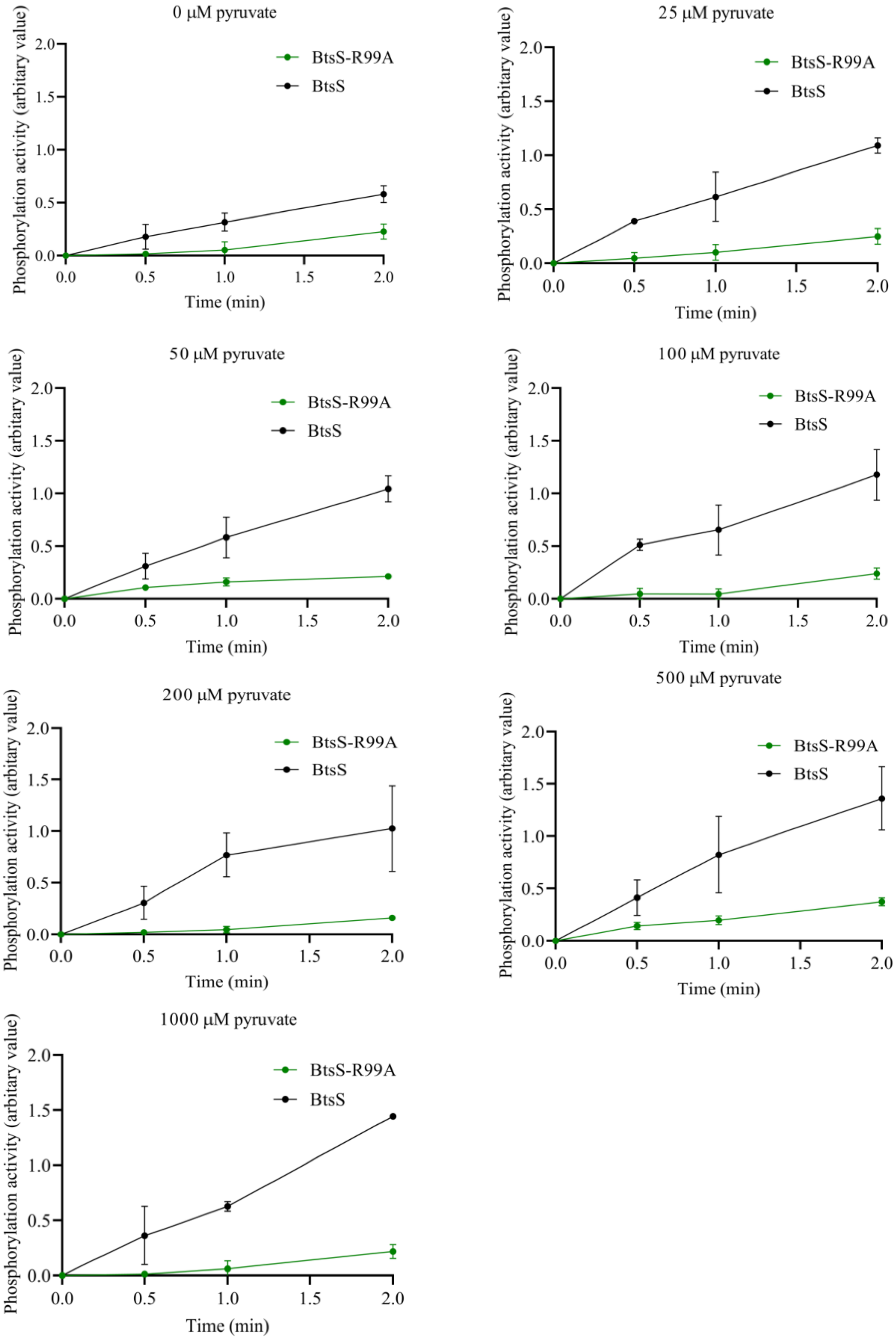

FIG S5 The influence of increasing pyruvate concentrations on the autophosphorylation activity of BtsS-R99A compared to wild-type BtsS. The same experimental approach as in FIG S4 was used.

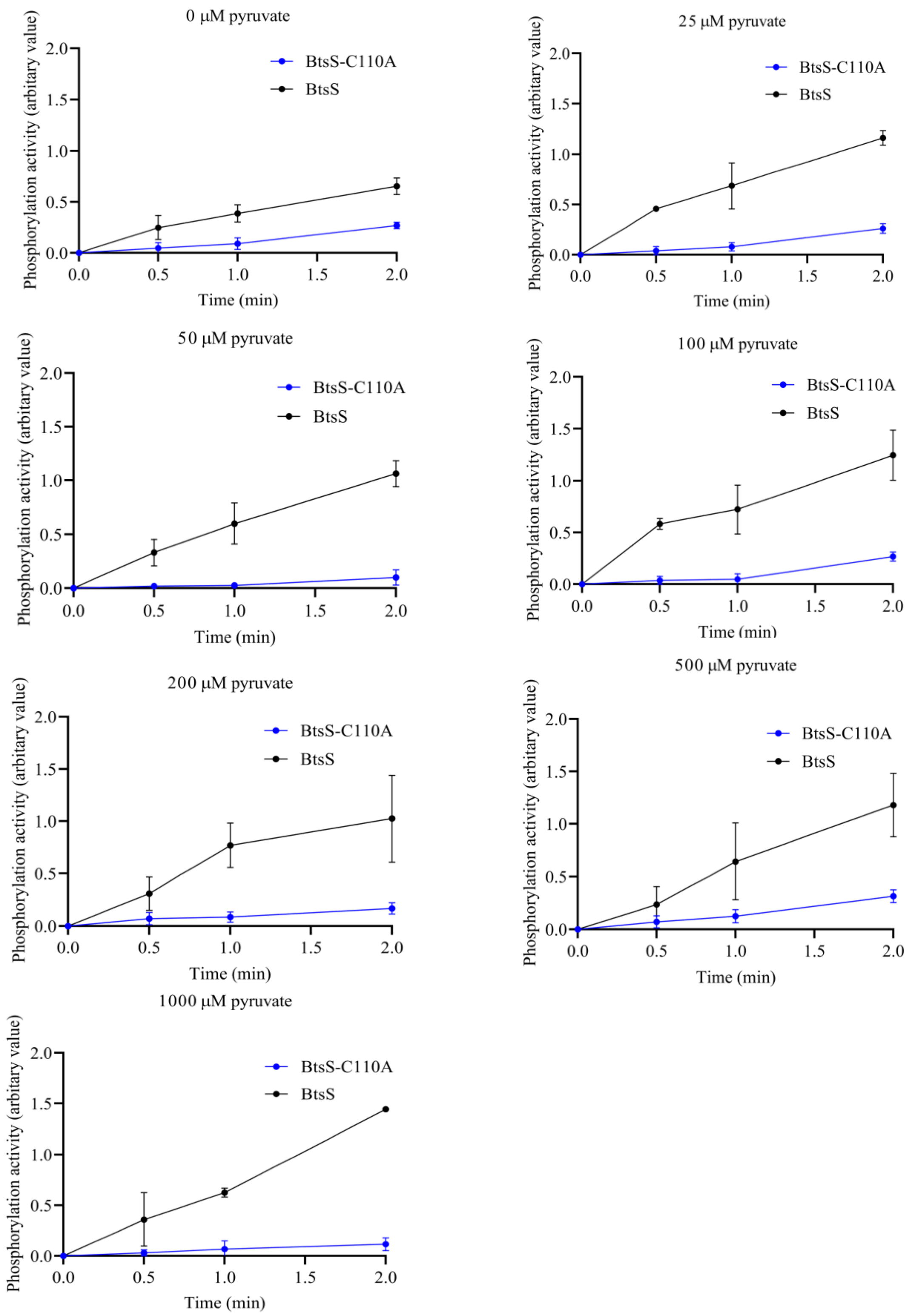

FIG S6 The influence of increasing pyruvate concentrations on the autophosphorylation activity of BtsS-C110A compared to wild-type BtsS. The same experimental approach as in FIG S4 was used.

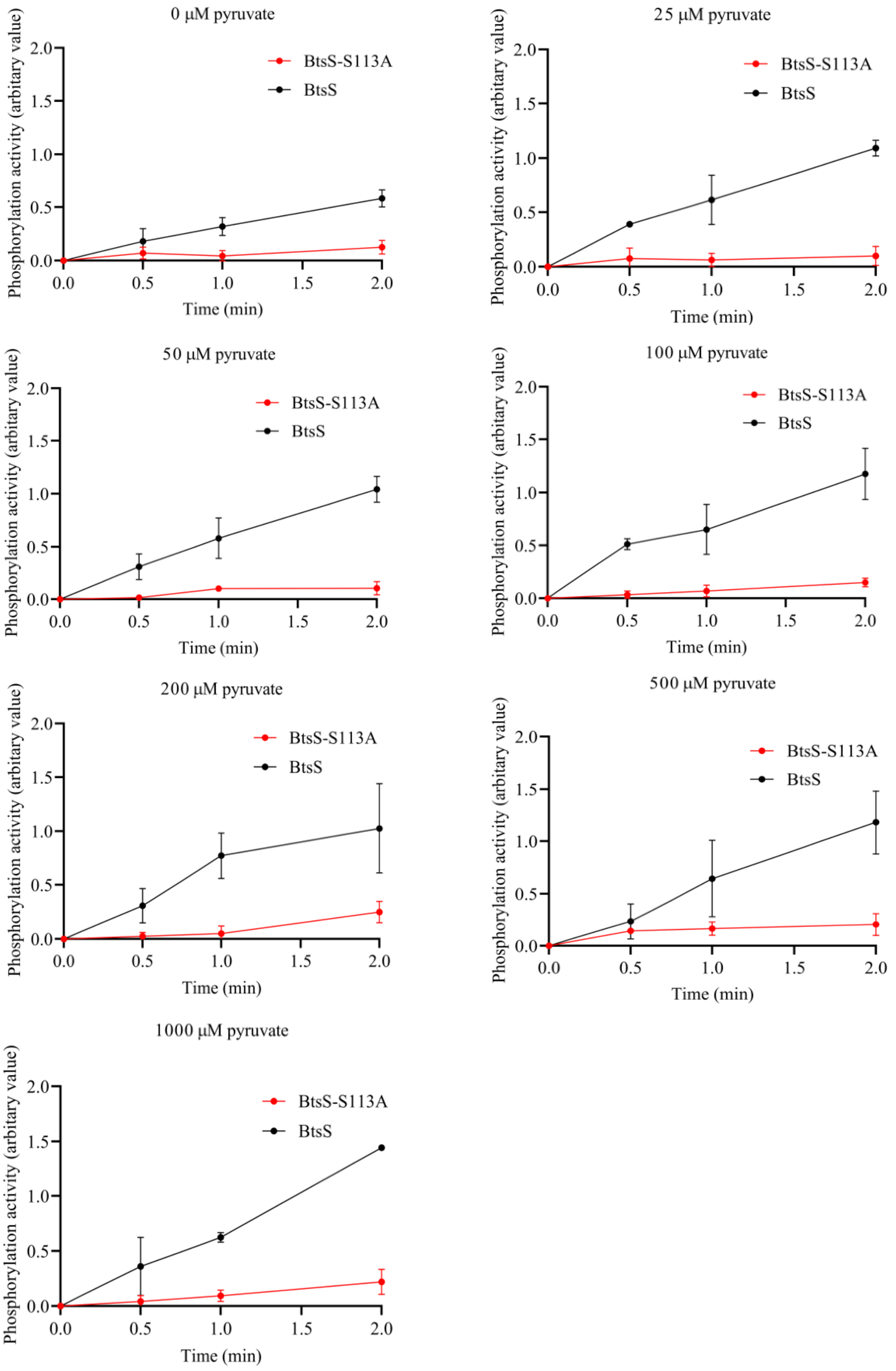

FIG S7 The influence of increasing pyruvate concentrations on the autophosphorylation activity of BtsS-S113A compared to wild-type BtsS. The same experimental approach as in FIG S4 was used.

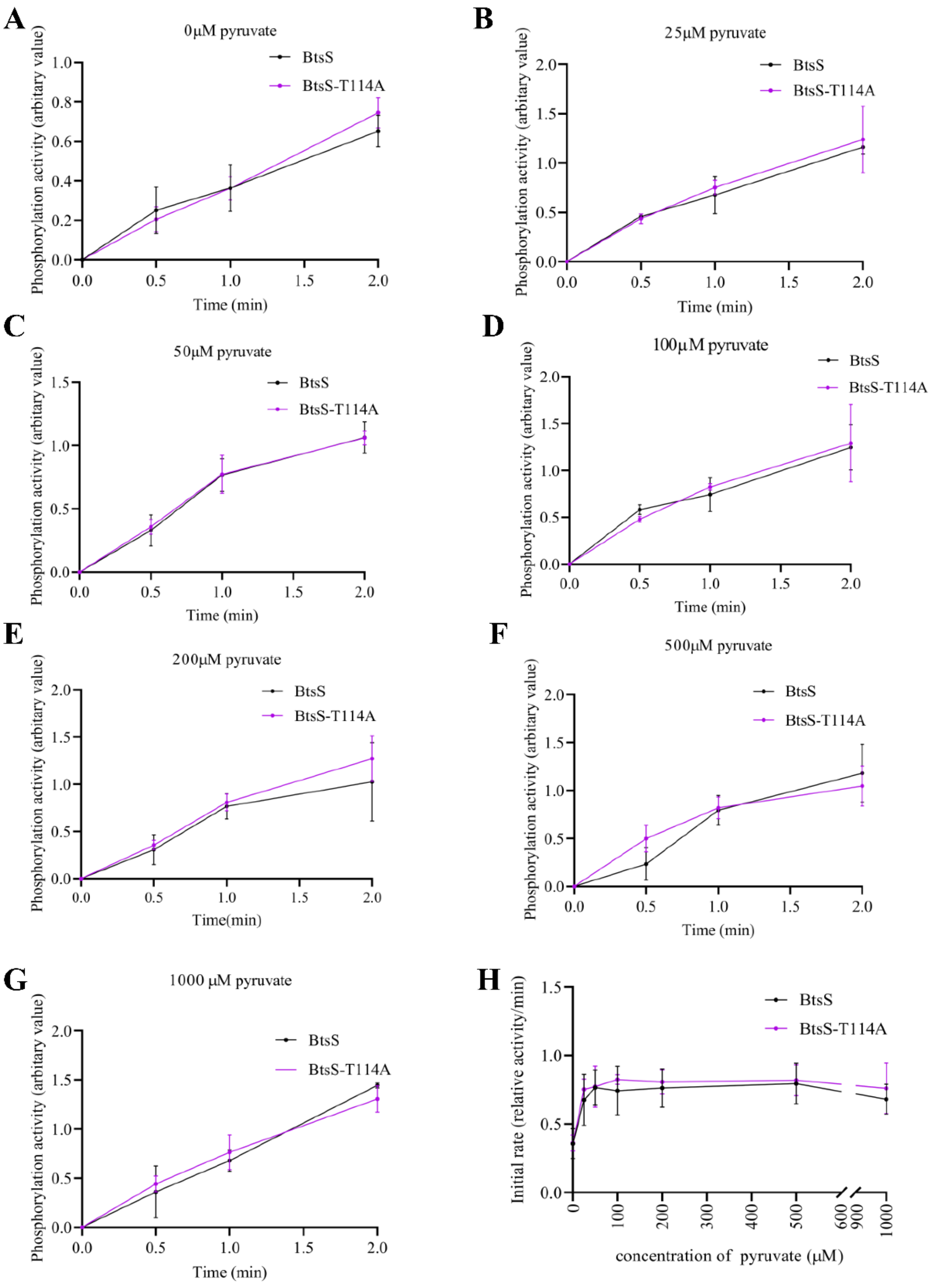

FIG S8 The influence of increasing pyruvate concentrations on the autophosphorylation activity of BtsS-T114A compared to wild-type BtsS. The same experimental approach as in FIG S4 was used. (A-G) Pyruvate concentration dependency of autokinase activity. (H) The effect of increasing pyruvate concentrations on the initial rate of autophosphorylation BtsS and BtsS-T114A.

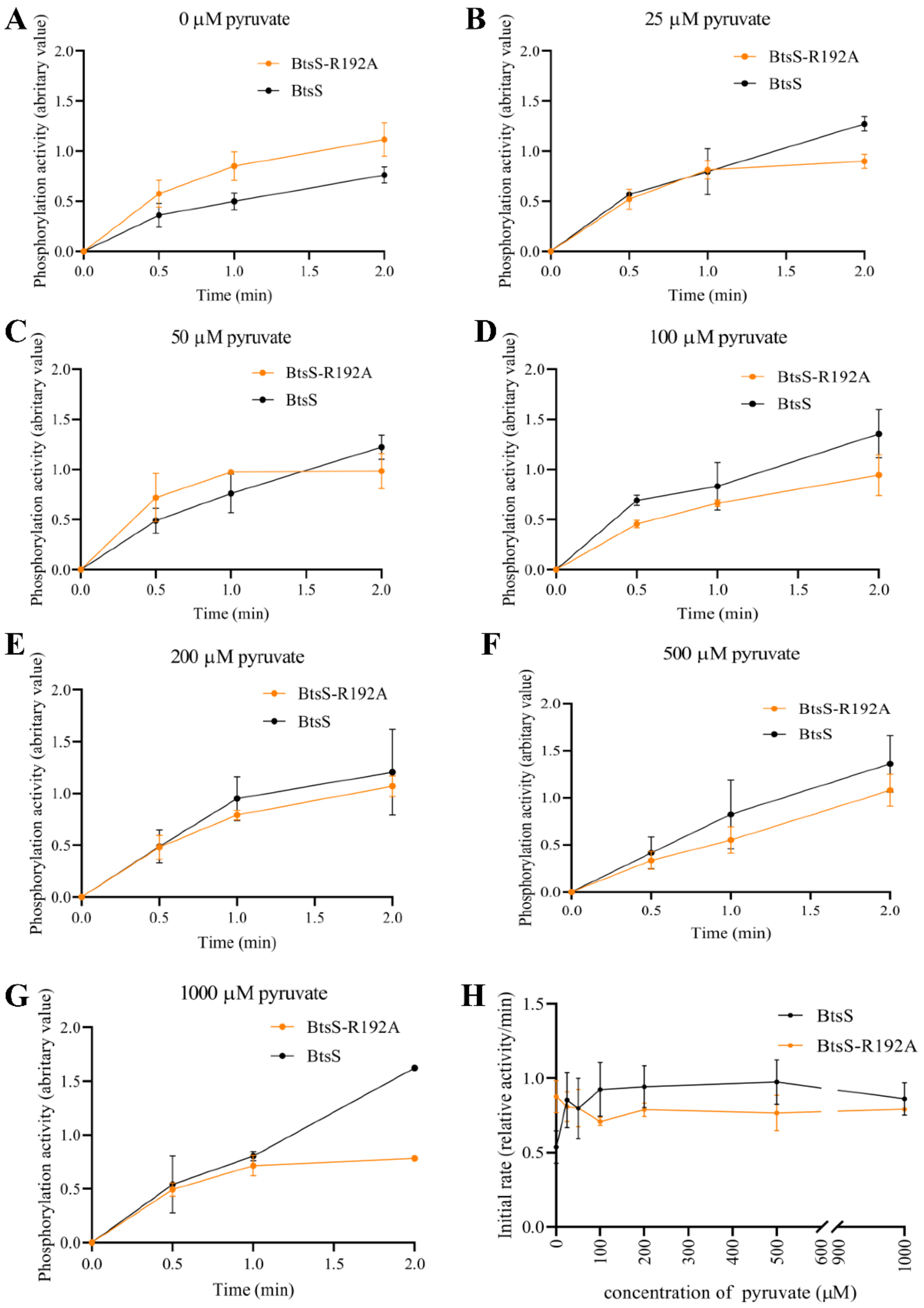

FIG S9 The influence of increasing pyruvate concentrations on the autophosphorylation activity of BtsS-R192A compared to wild-type BtsS. The same experimental approach as in FIG S4 was used. (A-G) Pyruvate concentration dependency of autokinase activity. (H) The effect of increasing pyruvate concentrations on the initial rate of autophosphorylation BtsS and BtsS-R192A.

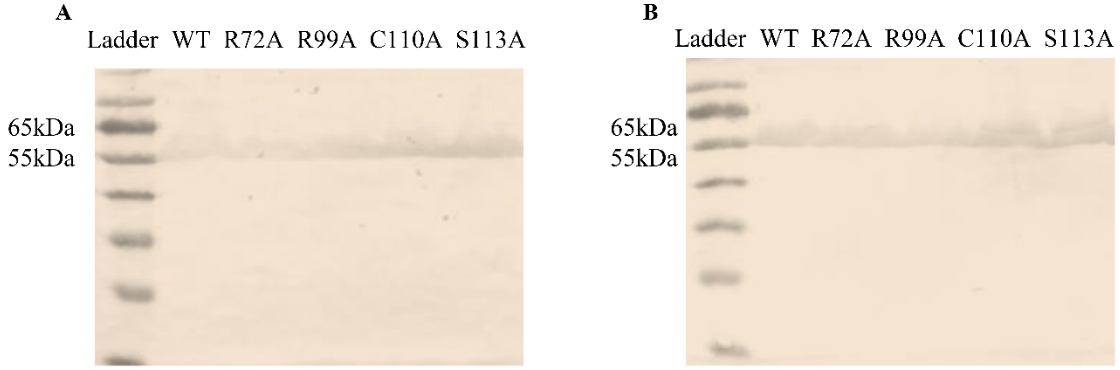

FIG S10 Western blot for the BtsS-6His-T18 or BtsS-6His-T25 hybrid proteins. Verification of production and integration of BtsS-T18 (A) and BtsS-T25 variants (B) in the cytoplasmic membrane of *E. coli* BTH101. Cells were disrupted and fractionated, 25 µg protein of the membrane fraction was analyzed by SDS-PAGE and Western blotting. BtsS was detected by a monoclonal mouse antibody against the His tag and an alkaline phosphatase-coupled secondary antibody.

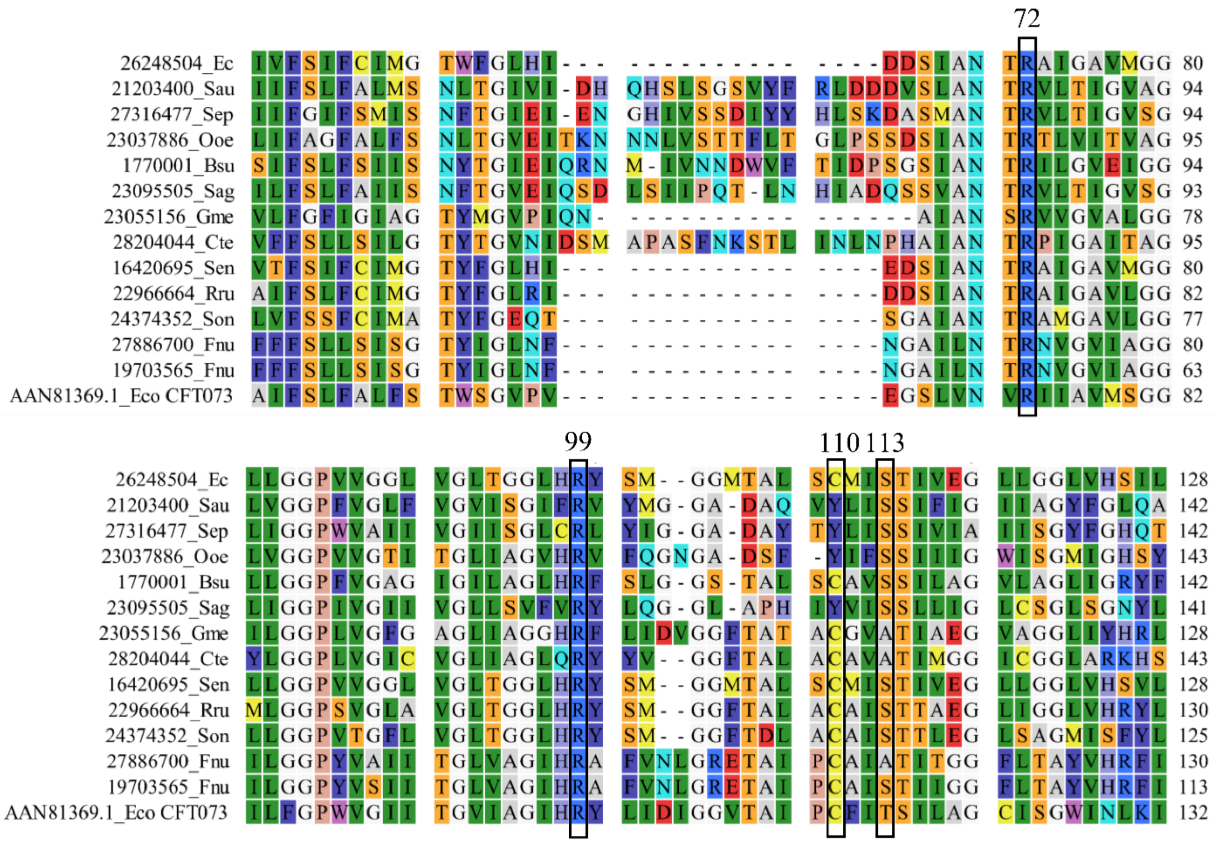

FIG S11 Multiple sequence alignment of BtsS with other LytS kinases. The species abbreviations are, Bsu-*Bacillus subtilis*; Cte-*Clostridium tetani*; Ec-*Escherichia coli*; Fnu-*Fusobacterium nucleatum*; Gme-*Geobacter metallireducens*; Ooe-*Oenococcus oeni*; Rru-*Rhodospirillum rubrum*; Sag-*Streptococcus agalactiae*; Sau-*Staphylococcus aureus*; Sen-*Salmonella enterica*; Son-*Shewanella oneidensis*; Sep-*Staphylococcus epidermidis*. Numbers correspond to GenBank (NIH) records. Positions corresponding to Arg72, Arg99, Cys110 and Ser113 of BtsS are framed with a black box.
